# FGL-1 plasma levels correlate with markers of inflammation and severe disease during SARS-CoV-2 infection

**DOI:** 10.1038/s43856-026-01775-4

**Published:** 2026-07-31

**Authors:** Sophia Cichutek, Leon Cords, Kilian Keetz, Marissa Herrmann, Liz J. Lam, Maria Mader, Robin Woost, Stefan Schmiedel, Ansgar W. Lohse, Carsten Bokemeyer, Christoph Schultheiß, Marylyn M. Addo, Walter Fiedler, Mascha Binder, Julian Schulze zur Wiesch

**Affiliations:** 1 Department of Oncology, Hematology and Bone Marrow Transplantation with Section Pneumonology, University Medical Center Hamburg-Eppendorf, Hamburg, Germany; 2 I. Department of Medicine, University Medical Center Hamburg-Eppendorf, Hamburg, Germany; 3 German Center for Infection Research (DZIF), Partner Site Hamburg-Lübeck-Borstel-Riems, Hamburg, Germany; 4 Division of Medical Oncology, University Hospital Basel, Basel, Switzerland; 5 Laboratory of Translational Immuno-Oncology, Department of Biomedicine, University and University Hospital Basel, Basel, Switzerland; 6 Institute for Infection Research and Vaccine Development (IIRVD), University Medical Center Hamburg-Eppendorf, Hamburg, Germany

**Keywords:** Viral infection, T cells

## Abstract

**Background:**

Fibrinogen-like protein 1 (FGL-1), primarily secreted by the liver, modulates immune responses and is upregulated in patients with hepatocellular carcinoma. FGL-1 affects T-cell function by interacting with lymphocyte-activation gene 3 (LAG-3), and blocking this interaction can enhance T-cell activity, making it a potential therapeutic target. In coronavirus disease 2019 (COVID-19), the upregulation of immune checkpoints on T cells, particularly LAG-3, is associated with disease severity. Liver inflammation and damage are well-known features of COVID-19 pathogenesis; however, little is known about FGL-1 expression patterns in COVID-19 and other infectious diseases.

**Methods:**

In a cohort of 65 patients with COVID-19 and 33 healthy donors, plasma FGL-1 levels were measured, and their correlations with clinical parameters and the course of the disease were evaluated. Additionally, both bulk and severe acute respiratory syndrome coronavirus type 2 (SARS-CoV-2)-specific T-cell immune phenotypes, as well as SARS-CoV-2-specific immune responses were comprehensively characterized and measured using multicolor flow cytometry and pMHC multimer technology.

**Results:**

Plasma FGL-1 levels were elevated in patients with COVID-19 and correlated with IL-6 and disease severity. LAG-3 was significantly upregulated on bulk and COVID-19-specific CD8^+^ T cells and was co-expressed with T-cell activation markers (CD38, HLA-DR) and inhibitory receptors (PD-1, TIGIT).

**Conclusion:**

These findings suggest a role for FGL-1 in the pathogenesis of COVID-19, potentially through modulation of T-cell activation and function via LAG-3, and may also be relevant in other hyperinflammatory conditions where immune modulation plays a role.

**Figure:**
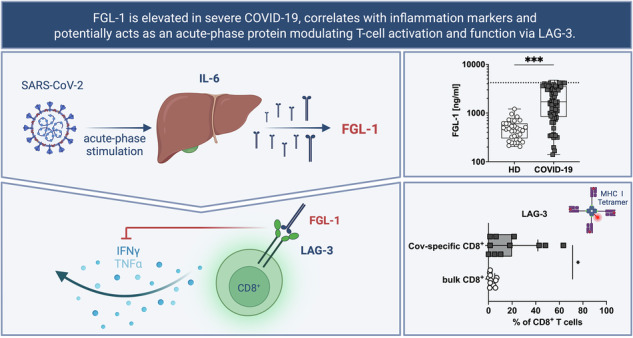

## Plain language summary

This study investigated how fibrinogen-like protein 1 (FGL-1) may be involved in COVID-19. FGL-1 is produced by the liver and can interact with the molecule LAG-3, which helps regulate the activity of immune cells. In severe COVID-19, the immune system can become unbalanced and less effective, especially certain immune cells called T cells. We analyzed blood samples from 65 patients with COVID-19 and 33 healthy individuals. Patients with COVID-19 had higher levels of FGL-1, and higher levels were linked with increased inflammation and more severe disease. In COVID-19 patients, T cells showed higher levels of LAG-3 and signs of strong immune activation. Overall, our findings suggest that the interaction between FGL-1 and LAG-3 may play an important role in how the immune system responds to COVID-19. This pathway could be a potential target for future treatments of COVID-19 and other diseases involving excessive inflammation.

## Abstract (web summary)

Cichutek et al. examine plasma FGL-1 and LAG-3 expression in COVID-19, alongside clinical and immune parameters. They find elevated FGL-1 associates with higher IL-6 concentrations and more severe disease, and increased LAG-3 expression on CD8⁺ T cells, pointing to a potential role of the FGL-1/LAG-3 axis in COVID-19-associated immune dysregulation.

## Introduction

Coronavirus disease 2019 (COVID-19), caused by severe acute respiratory syndrome coronavirus type 2 (SARS-CoV-2), presents with a broad spectrum of clinical manifestations, ranging from mild symptoms to acute respiratory distress syndrome (ARDS) and multi-organ failure. These severe outcomes are driven by dysregulated immune responses and hyperinflammatory states^1^. Key features of this pathophysiology include elevated pro-inflammatory cytokines, notably interleukin-6 (IL-6), and increased expression of inhibitory receptors such as T-cell immunoglobulin mucin-3 (TIM-3) and lymphocyte-activation gene 3 (LAG-3)^2,3^. As a hallmark mediator of systemic inflammation in severe COVID-19^4,5^. IL-6 has become a therapeutic target, and anti-IL-6 receptor antibodies (e.g., tocilizumab) have shown some effects in reducing inflammation, and in improving outcomes in critically ill patients^6,7^. Beyond pulmonary complications, systemic inflammation and IL-6-driven immune responses are associated with liver injury, a common clinical feature in COVID-19^8^. Liver inflammation is significantly more prevalent in severe cases and serves as a critical indicator of increased mortality^9–11^.

Fibrinogen-like protein 1 (FGL-1) is mainly secreted by the liver and plays a role in immune regulation and cellular protection^12,13^. It promotes cell growth and regeneration in response to liver injury^14,15^, and its expression in hepatocytes is upregulated in an IL-6-dependent manner^16^. Furthermore, FGL-1 expression is upregulated in various cancers, including hepatocellular carcinoma (HCC), and has been linked to T-cell exhaustion and reduced T-cell infiltration in the liver microenvironment^17–21^.

Recent studies have identified FGL-1 as a primary ligand for the immune checkpoint receptor LAG-3^22^. In cancer, high FGL-1 levels correlate with poor outcomes and resistance to immune checkpoint blockade therapies driven by immune evasion via LAG-3 engagement^22,23^. Recombinant FGL-1 protein has been reported to partially inhibit LAG-3-dependent T-cell activation, and specific blockade of the FGL-1/LAG-3 interaction restored T-cell function, further inhibiting tumor growth in vivo^22,24,25^. LAG-3 is a key marker of CD8^+^ T-cell exhaustion, upregulated across various malignancies and chronic viral infections^26–32^. In these settings, elevated LAG-3 expression impairs effector function, making its blockade a promising strategy for restoring immune responses^32,33^. Notably, LAG-3 inhibitors combined with anti-PD-1 therapy have already demonstrated improved efficacy in melanoma, highlighting the potential of this approach for both cancer and viral diseases^31,34^. While the role of LAG-3 in infectious diseases is well established, little is known about its ligand, FGL-1, in this context. The FGL-1/LAG-3 axis may be an intrinsic regulatory mechanism to prevent IL-6-mediated hyperinflammatory states. Inappropriate levels of FGL-1 secretion and the resulting dysregulation of the FGL-1/LAG-3 axis, however, could contribute to ineffective or even harmful immune responses observed in severe COVID-19^5^. Therefore, understanding the role of the FGL-1/LAG-3 axis in the immune response to COVID-19 can provide important insights into the mechanisms underlying the progression of severe disease and reveal potential therapeutic targets. This study aimed to analyze plasma FGL-1 concentrations in patients with COVID-19 and examine the phenotype of their lymphocyte populations, focusing on CD8^+^ T-cell immune phenotypes and SARS-CoV-2-specific immune responses.

In this observational, retrospective study, plasma FGL-1 levels and LAG-3 expression, and their association with disease severity, are analyzed in a cohort of 65 COVID-19 patients and 33 healthy donors. In addition, we comprehensively characterized both bulk and SARS-CoV-2-specific CD8^+^ T cell immune phenotypes to better understand T cell dysregulation in acute COVID-19. Plasma FGL-1 levels are elevated in COVID-19 and correlate with IL-6 levels and disease severity. LAG-3 is increased on bulk and SARS-CoV-2-specific CD8^+^ T cells and is co-expressed with activation and inhibitory markers. By advancing knowledge of the FGL-1/LAG-3 landscape in COVID-19 immune responses, this research provides a basis for further investigation of this axis in COVID-19 and possibly in other inflammatory diseases.

## Methods

### Patient cohort

The study participants were enrolled in this study between March 2020 and January 2021 at the University Medical Center Hamburg-Eppendorf, Germany. Venous whole blood samples from patients with SARS-CoV-2 (*n* = 65) were collected in Vacutainer Cell Preparation (CPT) tubes (BD Biosciences, New Jersey, USA). For comparison, we included 33 uninfected individuals (13 female, 20 male) without signs of infection and relevant comorbidities (Supplementary Table 1). Out of those individuals, 12 were unvaccinated; samples from the remaining 21 individuals were obtained after they had received a COVID-19 vaccination. Further, patients with hepatitis C virus (HCV) (*n* = 14) and human immunodeficiency virus-1 (HIV) infection (*n* = 35) served as the control group. All patients with COVID-19 were treated as inpatients, and SARS-CoV-2 infection was confirmed by polymerase chain reaction (PCR) of a nasopharyngeal or oropharyngeal swab specimen, as previously described^35^. Since the cohort included samples from the first and second waves of the COVID-19 pandemic, the viral strains were likely representative of the regionally dominant SARS-CoV-2 lineages at that time, namely B.1 and B.3^36^. Disease severity was graded based on clinical presentation according to the STAKOB classification (Robert Koch Institute, Berlin, Germany; adapted from the WHO Therapeutics and COVID-19: Living Guideline, Supplementary Table 2)^37,38^. Similar findings are reported for both sexes. All participants gave written informed consent. The study was performed in accordance with local legal requirements (§12 Hamburgisches Krankenhausgesetz) and approved by the Ethics Committee of the Medical Council of Hamburg (*Ärztekammer Hamburg:* PV4780, PV7298, WF14-09, PV4081, MC-316/14).

### Sample processing

Peripheral blood mononuclear cells (PBMC) were isolated by density gradient centrifugation and cryopreserved in Roswell Park Memorial Institute medium (RPMI1640, Gibco) supplemented with 25% heat-inactivated fetal calf serum and 10% dimethylsulfoxide at −180 °C. From the supernatant, aliquots of sodium citrate-anticoagulated plasma were stored separately at −80 °C. Cryopreserved PBMC or plasma samples were thawed as needed and used for subsequent applications as described below.

### ELISA

Plasma levels of FGL-1 and soluble LAG-3 (sLAG-3) were quantified by sandwich enzyme-linked immunosorbent assay (ELISA) using the Human FGL-1 ELISA Kit ab284622 (Abcam, Cambridge, UK) and Human LAG-3 ELISA Kit BMS2211 (Invitrogen, Thermo Fisher Scientific, Waltham, MA, USA). Assays were performed according to the manufacturer’s protocol. Plasma samples were diluted 1:1000 for FGL-1 and 1:10 for sLAG-3 in assay buffer, as determined by prior titration (Supplementary Fig. 1). Absorbance was measured at 450 nm using a microplate spectrophotometer. Concentrations were calculated based on a standard curve generated for each plate using a four-parameter logistic (4PL) curve fit. The detection range was 46.88−4200 ng/ml for FGL-1 and 6.2−400 pg/ml for sLAG-3.

### Multiparameter flow cytometry

For multiparametric flow cytometry analysis, cryopreserved PBMCs from patients with COVID-19 and healthy donors were thawed. After washing with PBS and FcR blocking, fluorochrome-conjugated surface antibodies (Supplementary Table 3, Panel 1) were added and incubated for 30 min. Dead cells were excluded by LIVE/DEAD Fixable Near-IR Dead Cell Stain (Invitrogen, Thermo Fisher Scientific, Waltham, MA, USA). Cells were fixed using 1% paraformaldehyde. Single-stained Comp Beads (BD Biosciences, New Jersey, USA) served as compensation controls. All samples were run on a BD FACSymphony A3 (BD Biosciences, New Jersey, USA). An exemplary gating strategy of immune cell phenotyping and representative dot plots of the LAG-3^+^, PD-1^+^, TIGIT^+^, CD38^+^/HLA-DR^+^, CD39^+^, and CD73^+^ CD8^+^ T cell population are demonstrated in Supplementary Figs. 2 and 3, respectively.

### HLA typing

High-definition molecular HLA typing was performed at the Institute of Transfusion Medicine at the University Medical Center Hamburg-Eppendorf by polymerase chain reaction sequence-specific oligonucleotide (PCR-SSO) using the commercial kit SSO LabType (One Lambda, Canoga Park, CA, USA).

### MHC class I tetramer staining

Cryopreserved PBMCs were thawed and stained with an APC-conjugated HLA-A*02:01 YLQPRTFLL (SARS-CoV-2 Spike 269-277) tetramer (provided by the NIH Tetramer Core Facility, Atlanta, GA, USA) for 30 min. The cells were then washed and stained with fluorochrome-conjugated surface antibodies as indicated in Supplementary Table 3 (Panel 2). After fixation with 1% paraformaldehyde, the cells were analyzed by flow cytometry using the BD FACSymphony A3 (BD Biosciences, New Jersey, USA). An exemplary gating strategy is demonstrated in Supplementary Fig. 4. Tetramer specificity was confirmed by the absence of tetramer-positive cells in three HLA-A*02:01-negative COVID-19 patients (Supplementary Fig. 5).

### Data and statistical analysis

All flow cytometric data were analyzed using FlowJo v10.8 software (BD Biosciences, New Jersey, USA). Graphs and plots were created using GraphPad Prism v10.3.1 (GraphPad Software Inc., San Diego, CA, USA). The statistical tests used to assess statistical significance in unpaired and paired datasets are indicated in the respective figure legends. Spearman’s rank correlation coefficient was calculated to determine the correlation between different data sets. Data are expressed as mean with standard deviation.

## Results

### Clinical characteristics of patients with acute SARS-CoV-2 infection

65 individuals hospitalized with acute SARS-CoV-2 infection were recruited at the University Medical Center Hamburg-Eppendorf between March 2020 and January 2021 (Table 1). None of the included patients had received COVID-19 vaccination. Infected patients had different clinical manifestations, with mild (*n* = 9), moderate (*n* = 23), severe (*n* = 22), or critical (*n* = 11) courses of COVID-19. The patients were enrolled within 30 days (mean = 7 days) after they tested positive for SARS-CoV-2 by PCR from nasopharyngeal or oropharyngeal swabs. As expected, all groups showed significant differences in terms of oxygen supplementation and intensive care unit (ICU) admission. All patients with a severe or critical course of disease (*n* = 33/33; 100%) required oxygen supplementation, whereas only 13.0% (*n* = 3/23) of patients with moderate disease received oxygen. Among individuals with moderate COVID-19, 7.7% (*n* = 1/23) were admitted to the ICU, but no invasive ventilation or extracorporeal membrane oxygenation (ECMO) was required. However, 45.5% (*n* = 10/22) of severely infected patients and 100% (*n* = 11/11) of critically infected patients required ICU admission, with 13.6% (*n* = 3/22) and 63.6% (*n* = 7/11) requiring invasive ventilation, respectively. ECMO was established in one patient (9.1%) with critical COVID-19. Mean values of systemic inflammation markers, such as C-reactive protein (CRP), IL-6, and Ferritin, increased with a more severe disease course. Levels of IL-6 were highest in critically ill patients compared to those with a severe, moderate, or mild course of disease (mean IL-6 = 28.2 ng/l vs. 19.1 ng/l vs. 18.8 ng/l and 5.5 ng/l, respectively).

**Table 1:** Clinical characteristics of patients with acute SARS-CoV-2 infection enrolled in the study

	COVID-19				*p*-value
	Mild *n* = 9	Moderate *n* = 23	Severe *n* = 22	Critical *n* = 11	
Age in years, mean (range)	44 (27–77)	59 (22–86)	65 (35–89)	65 (35–82)	0.091
Sex at birth, *n* (%)					
Female	6 (66.7%)	13 (56.5%)	6 (27.3%)	5 (45.5%)	0.128
Male	3 (33.3%)	10 (43.5%)	16 (72.7%)	6 (54.5%)	
Days since diagnosis, mean (range)	5.0 (1–21)	7.0 (1–17)	7.5 (1–36)	18 (2–50)	<0.001
Comorbidities, n (%)					
None	4 (44.4%)	3 (13.0%)	1 (4.5%)	‒	
Hypertension	1 (11.1%)	8 (34.8%)	10 (45.5%)	5 (45.5%)	
Diabetes	‒	5 (21.7%)	4 (18.2%)	5 (45.5%)	0.118
Heart diseases	2 (22.2%)	5 (21.7%)	9 (40.9%)	1 (9.1%)	
Lung diseases	1 (11.1%)	4 (17.4%)	4 (18.2%)	5 (45.5%)	
Cancer	‒	3 (13.0%)	7 (31.8%)	4 (36.4%)	
Other	2 (22.2%)	3 (13.0%)	4 (18.2%)	2 (18.2%)	
Intensive Care Unit, n (%)					
Invasive	‒	1 (7.7%)	10 (45.5%)	11 (100%)	
Ventilation	‒	‒	3 (13.6%)	7 (63.6%)	<0.001
ECMO	‒	‒	‒	1 (9.1%)	
Oxygen supplementation, *n* (%)	0 (0%)	3 (13.0%)	22 (100%)	11 (100%)	<0.001
Laboratory results, mean (range)					
IL-6 [ng/l]	5.5 (<1.5–15)	18.8 (2.7–372)	19.1 (2.7–211)	28.2 (7.3–231)	0.548
CRP [mg/l]	5 (<4–46)	44 (<4–197)	36 (4–240)	76 (4–207)	0.023
Ferritin [µg/l]	254 (205–392)	344 (16.7–2500)	1318 (272–3023)	551 (112–2487)	0.001
AST [U/l]	33 (14–70)	33 (13–138)	40.5 (13–122)	41 (10–107)	0.574
ALT [U/l]	23.5 (9–85)	30 (11–161)	36.5 (14–158)	35 (16–84)	0.001
LDH [U/l]	254 (168–318)	315 (244–673)	414 (192–813)	339 (244–467)	0.007
Lymphocytes [10^9^/l]	1.35 (0.8–1.9)	1.12 (0.3–3.42)	1.02 (<0.1–8.3)	0.88 (0.63–1.85)	0.588
D-dimer [mg/l]	0.8 (<0.2–1.5)	0.8 (0.3–3.5)	1.2 (0.37–4.1)	1.5 (0.6–3.7)	0.495
30-day mortality, *n* (%)	0 (0%)	0 (0%)	1 (4.6%)	2 (18.2%)	0.125

Values are presented as absolute numbers, percentages, or means with ranges. Time since diagnosis refers to the time elapsed since the first positive *PCR* swab result. Laboratory results were derived from the clinical database from the day of the sampling ±2 days. Abbreviations, *ECMO* extracorporeal membrane oxygenation, *CRP* C-reactive protein, IL-6, Interleukin-6, *AST* aspartate transaminase, *ALT* alanine transaminase, *LDH* lactate dehydrogenase. Reference values of laboratory results, *IL*-6 < 7 ng/l, *CRP* < 5 mg/l, Ferritin 10-291 µg/l, *AST* 10-35U/l, *ALT* 10-35U/l, *LDH* 0-200U/l, lymphocytes 1.1-3.4 × 10^9^/l, D-dimer 0.21–0.52 mg/l. The *p*-values were analyzed using the one-way ANOVA or Chi-square-test (≥0.05 (ns), <0.05 (*), <0.01 (**), < 0.001 (***)). The *p*-value for comorbidities was calculated by comparing individuals with no comorbidities to those with at least one comorbidity.

### FGL-1 plasma levels are increased in patients with COVID-19 and correlate with disease severity and markers of inflammation

**Fig. 1:**
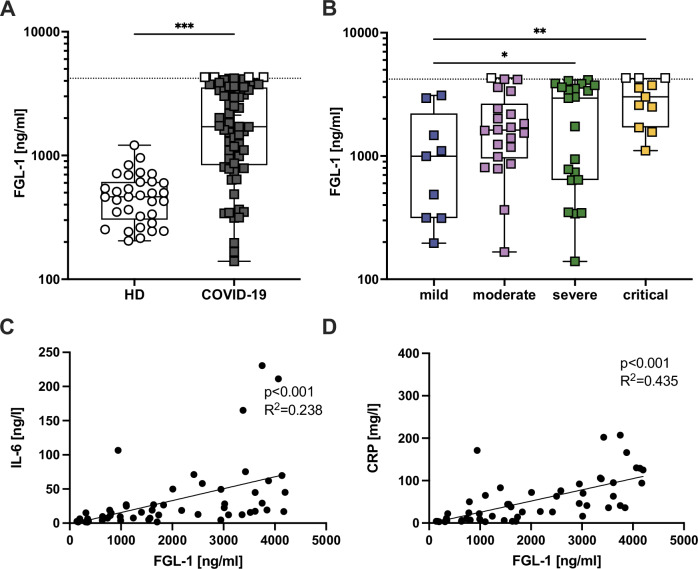
FGL-1 plasma levels are elevated in COVID-19 and correlated with disease severity and established markers of inflammation. **A** Plasma FGL-1 levels in patients with COVID-19 compared to healthy donors (HD). The dotted line represents the upper limit of detection in the ELISA (4200 ng/ml). White squares represent values above the upper limit of detection. A sample dilution of 1:1000 was used for the ELISA. **B** Correlation between plasma FGL-1 levels and the severity of COVID-19, as classified by the World Health Organization (WHO). **C** Correlation of plasma FGL-1 levels with Interleukin-6 (IL-6) and **D** C-reactive protein (CRP), respectively. The data were analyzed using the Mann–Whitney test (**A**) and Kruskal–Wallis test (**B**) (≥0.05 (ns), <0.05 (*), <0.01 (**), <0.001 (***)). Data are expressed as median with interquartile range. For bivariate correlation analyzes (**C**, **D**), the Spearman correlation was applied.

Severe COVID-19 cases are often associated with hyperinflammation characterized by elevated levels of pro-inflammatory cytokines, like IL-6^39,40^. Since FGL-1, similar to other acute-phase reactants, is a liver-derived secretory protein regulated by IL-6, we examined whether plasma FGL-1 levels were elevated in patients with COVID-19 using an ELISA^16^ (Fig. 1). Compared to healthy donors (mean, 492.9 ng/ml; SD, 229.9 ng/ml), mean plasma FGL-1 levels showed a four- to five-fold elevation in patients with COVID-19 (mean, 2115.9 ng/ml; SD, 1400 ng/ml; Fig. 1A). Among healthy donors, COVID-19 vaccination status was not associated with a significant difference in plasma FGL-1 levels (Supplementary Fig. 6). Furthermore, plasma FGL-1 levels were elevated in patients with COVID-19 compared to those with chronic viral infections, including HCV and HIV infection (Supplementary Fig. 7). Next, we analyzed the association between FGL-1 plasma levels and clinical parameters. Indeed, FGL-1 levels correlated with disease severity as classified by the World Health Organization (WHO), with significantly higher concentrations observed in patients with severe and critical disease compared to those with mild disease (Fig. 1B).

It has been demonstrated that IL-6 can induce FGL-1 expression in hepatocytes, suggesting a connection between systemic inflammation and increased FGL-1 plasma levels^16^. Consistent with these findings, our in vitro data using the hepatocyte cell line HepG2 as a liver model confirmed IL-6-mediated activation of STAT3 signaling along with increased secretion of FGL-1, supporting a link between inflammatory signaling and hepatic FGL-1 production (Supplementary Fig. 8). In this study, a moderate correlation was observed between FGL-1 and IL-6 levels **(**Fig. 1C). Also, C-reactive protein (CRP), a known prognostic marker in patients with COVID-19, showed a statistically significant but weak positive correlation with FGL-1 levels (Fig. 1D). Furthermore, the correlation analysis revealed a statistically significant relationship between FGL-1 plasma levels and LDH, a marker of cellular damage (*p* = 0.02) as well as a negative trend with lymphocyte count (*p* = 0.002) (Supplementary Fig. 9A, B), both of which are known biomarkers for the severity of COVID-19^11,41^. However, the coefficients of determination were low (LDH, *R*² =  0.10; lymphocyte count, *R*² = 0.185), indicating that FGL-1 plasma levels only account for a small part of the variability in LDH levels and lymphocyte counts. Ferritin levels, which typically are elevated as part of an acute-phase reaction, did not show a significant association with FGL-1 plasma levels (*p* = 0.1; Supplementary Fig. 9C). The liver enzymes aspartate transaminase (AST) and alanine transaminase (ALT), which are routinely used to assess hepatocyte damage, are known to be increased in the early phase of critical and lethal COVID-19, serving as an early marker of a complicated disease^11^. Within the COVID-19 cohort, 44.6% (*n* = 29/65) of patients exhibited elevated liver enzymes; this included 54.5% (*n* = 12/22) of those with severe and 81.8% (*n* = 9/11) of those with critical disease (Table 1). Based on this, we investigated the potential relationship between FGL-1 plasma levels and COVID-19-associated liver injury. However, our analysis revealed no significant correlation between plasma FGL-1 levels and AST or ALT (Supplementary Fig. 9D). In a multivariate model, only CRP remained significantly associated with FGL-1 levels (*β* = 10.03, 95% CI 3.94−16.12, *p* = 0.0018). None of the other clinical or laboratory parameters examined showed a statistically significant independent association with FGL-1 levels. However, the impact of disease severity might need further investigation in larger cohorts, as there is a possible trend toward higher FGL-1 levels with increasing disease severity (Supplementary Table 4).

Taken together, these results suggest that FGL-1 is an acute-phase protein in IL-6-mediated systemic inflammation during SARS-CoV-2 infection. Plasma FGL-1 levels correlated with CRP levels and showed a trend toward higher levels with greater disease severity in patients with COVID-19, suggesting that FGL-1 may be associated with systemic inflammation during acute SARS-CoV-2 infection.

### Longitudinal analysis of FGL-1 plasma levels allows monitoring of inflammation in severe COVID-19

**Fig. 2:**
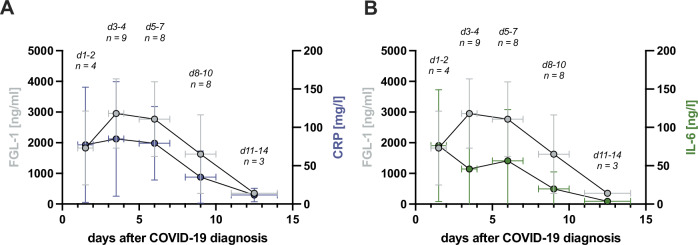
Longitudinal assessment of FGL-1 plasma levels and parameters of systemic inflammation in patients with severe and critical COVID-19. *N* = 32 patients with severe and critical COVID-19 with one measurement each were included in the analysis, and values for defined intervals were pooled (day 1–2, day 3–4, day 5–7, day 8–10, and day 11–14 since COVID-19 diagnosis). **A** Temporal course of plasma FGL-1 levels (grey) and CRP levels (blue) during SARS-CoV-2 infection. **B** Temporal course of plasma FGL-1 levels (grey) and IL-6 levels (green) during SARS-CoV-2 infection. CRP C-reactive protein, IL-6 Interleukin-6.

To investigate how plasma FGL-1 levels change over time, we tracked FGL-1 during SARS-CoV-2 infection. The relationship to established inflammation markers was assessed by interpolating separate curves for FGL-1 and CRP levels (Fig. 2A) and for FGL-1 and IL-6 (Fig. 2B), measured during the first 14 days after the initial diagnosis. Only 32 patients with severe or critical COVID-19, each with one measurement, were included in the analysis, and values for defined intervals were pooled (day 1–2, day 3-4, day 5–7, day 8–10, and day 11–14 since the first SARS-CoV-2-positive PCR result). After an increase early during SARS-CoV-2 infection, with an average peak 3–4 days after diagnosis, FGL-1 plasma levels decreased as the infection resolved and returned to near-normal values by day 10. The kinetics of plasma FGL-1 levels closely mimicked those of CRP. To further assess the ability of FGL-1, CRP, and IL-6 to distinguish between patients with mild versus moderate, severe, or critical COVID-19, receiver operating characteristic (ROC) curve analysis was conducted. FGL-1 showed significant discriminative ability (AUC = 0.7381, *p* = 0.0227). Although CRP and IL-6 demonstrated higher diagnostic accuracy in concurrent measurements (CRP AUC = 0.8460; IL-6 AUC = 0.8253), their performance increased further when peak levels were analyzed (CRP peak AUC = 0.8873; IL-6 peak AUC = 0.8948) (Supplementary Fig. 10). Since patient samples for FGL-1 analysis were not consistently collected at peak disease severity, the AUC observed likely underestimates its true discriminative ability.

Further, we analyzed longitudinal data on a case of prolonged SARS-CoV-2 infection. The clinical course and laboratory findings are presented in the supplementary material (Supplementary Fig. 11). We investigated plasma FGL-1 concentrations in this patient during COVID-19 disease on days 20, 26, 34, and 50 after COVID-19 diagnosis. We hypothesize that FGL-1 acts as an acute-phase protein, elevated during SARS-CoV-2 infection, and its plasma levels correlate with hyperinflammation. We therefore analyzed the association and course of plasma FGL-1 levels, markers of inflammation (Supplementary Fig. 11A), and liver enzymes (Supplementary Fig. 11B) in this patient with severe COVID-19 disease. Increased FGL-1 levels were associated with elevations of the markers of acute inflammation, including CRP, ferritin, and IL-6. After the initial increase early in SARS-CoV-2 infection, with a peak 36 days after the onset of symptoms, FGL-1 plasma levels fell as inflammation resolved and returned to a near-baseline value by day 50. Therefore, the kinetics of plasma FGL-1 levels closely resembled those of CRP and IL-6. Similarly, the liver enzymes AST and ALT showed a mild elevation, with a first peak 29 days after diagnosis, and normalized by day 50.

Overall, these data show high FGL-1 levels during acute systemic inflammation in COVID-19. The similar dynamics of IL-6 and CRP support its role as an acute-phase protein. However, larger cohorts and prospective approaches are needed to validate this association.

### Increased LAG-3 expression on CD8^±^ T cells in patients with COVID-19 is associated with markers of activation and immune exhaustion

FGL-1 was recently identified as a ligand for LAG-3^22^. In COVID-19, hyperactivated T effector cells exhibit increased frequencies not only of LAG-3 but also other co-inhibitory receptors such as PD-1 and TIGIT^2^. In cancer, the interaction of FGL-1 with its receptor LAG-3 was shown to have immunomodulatory properties^22,42^. During SARS-CoV-2 infection, a modulation of immune responses via the FGL-1/LAG-3 pathway could potentially contribute to immune dysfunction. To further understand the modulation of the FGL-1/LAG-3 axis, we analyzed immune cell subsets in 27 patients with COVID-19, four with mild, 10 with moderate, six with severe, and seven with critical disease, and compared them with those of 14 healthy individuals (study cohort details are presented in Supplementary Table 5). Previously, a generalized decrease in the T lymphocyte counts affecting CD8^+^ T cells has been described during SARS-CoV-2 infection^11^. In accordance with these results, we found significantly reduced CD8^+^ frequencies among CD3^+^ T cells in patients with COVID-19 (Supplementary Fig. 12A). Further, significantly lower CD8^+^ T-cell frequencies were observed in severe and critical COVID-19 disease compared to mild and moderate disease (Supplementary Fig. 12B, C).

**Fig. 3:**
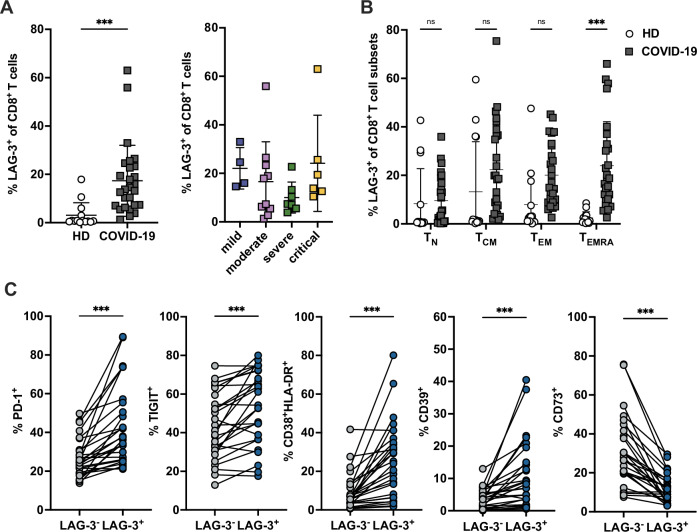
High frequency of LAG-3-positive CD8^+^ T cells in patients with COVID-19 compared to that of healthy donors (HD) is associated with markers of immune exhaustion and immune activation. **A** Frequencies of LAG-3^+^ CD8^+^ T cells in patients with COVID-19 and healthy donors (HD). **B** Frequency of LAG-3^+^ cells within different memory compartments of CD8^+^ T cells in patients with COVID-19 and HD. **C** Expression of PD-1, TIGIT, CD38/HLA-DR, CD39, and CD73 in LAG-3^+^ and LAG-3^-^ CD8^+^ T cells in patients with COVID-19. T_N_, naïve; T_CM_, central memory; T_EM_, effector memory; T_EMRA_, terminally differentiated effector memory cells. Unpaired groups (**A**, **B**) were analyzed using the Mann–Whitney and one-way ANOVA test, while paired groups (**C**) were analyzed with the Wilcoxon matched-rank test (≥0.05 (ns), <0.05 (*), <0.01 (**), <0.001 (***)). Data are expressed as median with interquartile range.

To confirm the increased LAG-3 expression on different immune cell subsets during acute SARS-CoV-2 infection in our cohort, we analyzed the frequencies of LAG-3-positive cells. Patients with COVID-19 showed a trend towards increased frequencies of LAG-3^+^ cells among all immune cell subsets compared with healthy donors (HD) (Supplementary Figs. 13 and 14A). Importantly, a significantly higher frequency of LAG-3^+^ cells was observed in the CD8^+^ T-cell compartment of patients with COVID-19 compared to HD (mean 24.92% vs. 9.36%, *p* = 0.01). The expression of LAG-3 on CD8^+^ T cells was comparable between patients with mild, moderate, severe, and critical COVID-19 (Fig. 3A). Although receptor shedding by immune cells could potentially increase plasma levels of soluble LAG-3 in patients with COVID-19, there was also no relevant difference compared to healthy donors (Supplementary Fig. 14B). Interestingly, despite both elevated frequencies of LAG-3^+^ immune cells and elevated plasma FGL-1 levels in SARS-CoV-2 infection, there was no correlation of plasma FGL-1 with the frequency of LAG-3^+^ cells of any of the analyzed immune subsets, nor with soluble LAG-3 in plasma samples (Supplementary Fig. 15). This may indicate that FGL-1 and LAG-3 expression are controlled by independent regulatory pathways.

Based on the differentiation markers CD45RA and CD27, naïve (T_N_) (CD45RA^+^ CD27^+^), central memory (T_CM_) (CD45RA^-^ CD27^+^), effector memory (T_EM_) (CD45RA^-^ CD27^-^), and terminal effector memory (T_EMRA_) (CD45RA^+^ CD27^-^) CD8^+^ T cells were defined and assessed for LAG-3 expression. LAG-3 was more frequently expressed on the T_EMRA_ CD8^+^ T-cell subset in patients with COVID-19 compared to HD (Fig. 3B). Moreover, in COVID-19 there was a shift towards late-differentiated CD8^+^ T cells, with a pronounced increase in CD8^+^ T_EMRA_ cells and a corresponding reduction in CD8^+^ T_N_ and T_CM_ cells (Supplementary Fig. 16). Consequently, the combination of the relative expansion of CD8^+^ T_EMRA_ cells and high LAG-3 expression within this subset resulted in an increased overall frequency of LAG-3^+^ CD8^+^ T cells.

To further characterize LAG-3^+^ CD8^+^ T cells, we analyzed the expression patterns of phenotypic activation markers and co-inhibitory receptors, particularly the co-expression of LAG-3/PD-1 and LAG-3/TIGIT on CD8^+^ T cells in patients with COVID-19 compared to HD. LAG-3^+^ CD8^+^ T cells showed significantly higher expression of PD-1, TIGIT, and CD39, and a decreased frequency of CD73^+^ cells compared to LAG-3^-^ CD8^+^ T cells in patients with COVID-19 (Fig. 3C). Notably, LAG-3^+^ CD8^+^ T cells also exhibited significantly higher co-expression of CD38 and HLA-DR (Fig. 3C).

Overall, we observed higher frequencies of LAG-3-positive cells, particularly within the CD8^+^ T_EMRA_ population, in COVID-19 patients. CD8^+^ effector T cells frequently co-expressed LAG-3 with PD-1, TIGIT, and CD39, alongside elevated activation markers (HLA-DR/CD38). Together, these data indicate robust effector T-cell activation and suggest a potential involvement of LAG-3^+^ CD8^+^ T cells in the acute immune response to SARS-CoV-2.

### SARS-CoV-2-specific CD8^+^ T cells show high expression of LAG-3 together with other co-inhibitory receptors and activation markers

Next, we aimed to investigate whether the activated phenotypes of bulk CD8^+^ T cells during COVID-19 arise from generalized immune activation, potentially induced by cytokines affecting both SARS-CoV-2-specific and bulk CD8^+^ T cells, or whether this is instead driven by the antigen-specific activation of SARS-CoV-2-specific CD8^+^ T cells. Therefore, we analyzed PBMCs of 11 HLA-A*02:01-positive patients (five female and six male, with a mean age of 58.3 years) with acute SARS-CoV-2 infection using an MHC class I tetramer specific for the SARS-CoV-2 S 269-277 epitope. Five patients presented with moderate, four with severe, and three with critical COVID-19 disease. At the time of blood collection, the average number of days since the initial diagnosis was 15.9 days. A detailed description of patient characteristics and relevant clinical parameters is given in Supplementary Table 6.

**Fig. 4:**
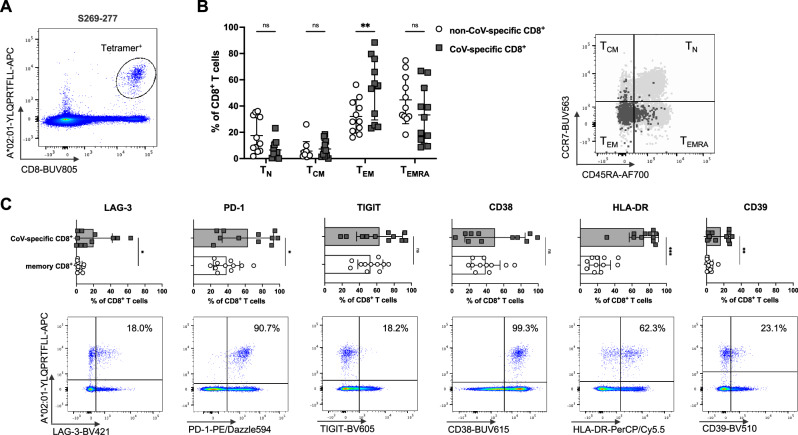
Ex vivo phenotyping of SARS-CoV-2-specific CD8^+^ T cells compared to non-SARS-CoV-2-specific CD8^+^ memory T cells from patients with COVID-19 (*n* = 11). **A** Representative HLA-A*02:01 tetramer staining (gated on live CD3^+^ T cells). **B** Frequency of naïve T_N_ (CCR7^+^/CD45RA^+^), central memory T_CM_ (CCR7^+^/CD45RA^-^), effector memory T_EM_ (CCR7^-^/CD45RA^-^), and terminally differentiated effector memory T_EMRA_ (CCR7^-^/CD45RA^+^) SARS-CoV-2-specific and non-SARS-CoV-2-specific (tetramer-negative) CD8^+^ memory T cells in patients with COVID-19. **C** Frequencies of LAG-3^+^, PD-1^+^, TIGIT^+^, CD38^+^, HLA-DR^+^, and CD39^+^ cells of all SARS-CoV-2-specific CD8^+^ T cells (grey bar) and non-SARS-CoV-2-specific CD8^+^ memory T cells (white bar) in patients with COVID-19. Representative dot plots (gated on CD8^+^ T cells) showing the phenotype of memory CD8^+^ T cells in patients with COVID-19. Percentages indicate the frequency of cells positive for each marker within the tetramer-positive population. For unpaired groups (**B**), statistical significance was assessed using the Mann–Whitney U test; for paired groups (**C**), the Wilcoxon matched-rank test was applied (≥0.05 (ns), <0.05 (*), <0.01 (**), <0.001 (***). Data are expressed as median with interquartile range.

Tetramer-positive cells were detected in all patients analyzed (Fig. 4A). CD8^+^ T-cell subsets were defined using established markers^43^: naïve T_N_ (CCR7^+^/CD45RA^+^), central memory T_CM_ (CCR7^+^/CD45RA^-^), effector memory T_EM_ (CCR7^-^/CD45RA^-^), and terminally differentiated effector memory T_EMRA_ (CCR7^-^/CD45RA^+^). As SARS-CoV-2-specific CD8^+^ T cells reside mainly within the memory compartment (i.e., non-naïve), we compared subset frequencies between SARS-CoV-2-specific and non-specific (tetramer-negative) memory populations. SARS-CoV-2-specific CD8^+^ T cells primarily belonged to the T_EM_ and T_EMRA_ compartment, with a significantly higher frequency of T_EM_ cells compared to non-specific memory CD8^+^ T cells (Fig. 4B). We also investigated possible correlations between ex vivo frequency of SARS-CoV-2-specific T cells and their LAG-3 expression with clinical parameters of COVID-19 or systemic inflammation (IL-6, CRP, FGL-1). However, in this small cohort, no significant correlations were observed (Supplementary Fig. 17).

To further characterize the activation status of SARS-CoV-2-specific CD8^+^ T cells, the expression of activation markers (CD38, HLA-DR, CD39), and co-inhibitory receptors (LAG-3, PD-1, and TIGIT) was assessed and compared to non-SARS-CoV-2-specific memory CD8^+^ T cells (Fig. 4C). SARS-CoV-2-specific CD8^+^ T cells showed significantly higher frequencies of LAG-3 expression (mean 19.9%, range 0–63.4%) than non-SARS-CoV-2-specific memory CD8^+^ T cells (mean 2.9%, range 1.0–7.0%). Notably, three patients (TET-02, TET-04, TET-08), lacked LAG-3 expression in both populations (Supplementary Table 6). Similarly, PD-1 and TIGIT also showed higher relative expression levels on SARS-CoV-2-specific CD8^+^ T cells compared to non-SARS-CoV-2-specific memory CD8^+^ T cells in all patients (mean 63.1%, range 23.1–97.9% vs. mean 31.6%, range 15.8–48.6%; mean 63.1%, range 18.2-93.5% vs. mean 44.7%, range 20.9–68.1%). Due to the limited sample size, no statistical significance was reached when stratifying by disease severity (Supplementary Fig. 18).

Activation markers were also elevated in the virus-specific compartment. HLA-DR showed the highest frequency (mean of 73.8% [range: 30.8–87.7%] vs. 18.7% [4.9–37.5%]), followed by CD38 (49.9% [3.9–99.3%] vs. 32.0% [18.9–52.9%]) and CD39 (16.3% [1.8–29.5%] vs. 2.7% [0.2–9.6]).

In conclusion, LAG-3 expression and the expression levels of other co-inhibitory receptors and activation markers were even higher on SARS-CoV-2-specific CD8^+^ T cells compared to non-SARS-CoV-2-specific memory CD8^+^ T cells in acute COVID-19. Hence, this is evidence that the overall highly activated phenotype of CD8^+^ T cells in SARS-CoV-2 infection is at least in part determined by the SARS-CoV-2-specific immune response.

## Discussion

In our study, patients with COVID-19 had significantly higher plasma levels of FGL-1 compared to healthy donors. These FGL-1 levels showed a strong positive correlation with disease severity and were associated with markers of acute inflammation, including CRP and IL-6. These findings are consistent with in vitro studies demonstrating that IL-6 directly upregulates FGL-1 expression in human hepatocytes via JAK2/STAT3-mediated signaling, suggesting a mechanistic link between the inflammatory cytokine response and hepatic FGL-1 production, similar to that of acute-phase reactants^16,23,44–46^. However, its properties and functional dynamics require further investigation through prospective studies. We found that the liver enzymes AST and ALT, which are surrogate markers for hepatocellular damage, were elevated in severe and critical COVID-19, even though they did not show a direct positive correlation with the FGL-1 plasma levels in our cohort. However, in a patient with severe disease, the kinetics of plasma FGL-1 levels showed an association with those of AST and ALT. After recovering from severe illness, the liver enzymes returned to normal levels over time. These findings are in line with previous studies reporting SARS-CoV-2-induced liver injury and underscore the liver’s role as both a target and a source of inflammatory mediators, including FGL-1^11,24,47^. Although the exact mechanisms, whether involving direct viral cytotoxicity or immune cell-mediated injury, have to be further investigated, our data support an association between liver inflammation and higher FGL-1 levels during severe COVID-19. However, these findings are observational and should be interpreted cautiously, as further studies are needed to clarify a potential functional role of the FGL-1/LAG-3 axis in COVID-19 pathogenesis.

FGL-1 may not only be associated with systemic inflammation but also contribute to its control through immunomodulatory effects, mediated by the engagement of its inhibitory receptor, LAG-3. Severe COVID-19 disease is characterized by immune dysregulation, including abnormal T-cell activation^48^. We and others observed increased frequencies of LAG-3-expressing CD8^+^ T cells, which co-expressed markers of activation such as PD-1, TIGIT, CD38, and HLA-DR^2^. Furthermore, more severe disease courses in patients with COVID-19 are associated with higher levels of these co-inhibitory receptors on CD8^+^ T cells compared to those with milder disease^2^, although these results could not be reproduced in this study, most likely due to an underpowered analysis.

Whether the observed phenotype of LAG-3^+^ CD8^+^ T cells arises from antigen-specific stimulation or from bystander activation driven by systemic cytokines remains unclear. Prior studies have described SARS-CoV-2-specific CD8^+^ T cells as dysfunctional due to reduced cytokine production yet displaying high levels of activation and co-inhibitory receptors, particularly in patients with severe disease^49^. However, Shahbaz et al.^50^ showed that overexpression of co-inhibitory receptors on SARS-CoV-2-specific CD8^+^ T cells reflected an activated rather than exhausted T cell phenotype, suggesting that immune dysregulation in COVID-19 may affect both specific and non-specific T-cell subsets. In line with this, our ex vivo analysis of SARS-CoV-2-specific CD8^+^ T cells predominantly showed an effector memory phenotype of these cells, with significantly higher expression of the co-inhibitory receptors LAG-3, PD-1, and TIGIT, as well as the activation markers CD38 and HLA-DR, compared to non- SARS-CoV-2-specific memory CD8^+^ T cells in patients with COVID-19. These findings are consistent with reports of hyperactivated, yet dysregulated, T-cell responses in COVID-19, which contribute to tissue damage and impaired viral clearance^5^.

IL-6-driven systemic inflammation in severe COVID-19 contributes to T-cell activation and immune cell dysfunction, characterized by the expression of co-inhibitory receptors, such as LAG-3^51^, which aligns with our findings. Lee et al.^52^ identified two groups of patients with severe to critical COVID-19 based on IL-6 levels, with the high IL-6 group displaying higher pro-inflammatory cytokines and T-cell activation markers, indicating distinct immune responses and possible T-cell exhaustion. In our cohort, patients with severe COVID-19 also presented with high IL-6 levels and SARS-CoV-2-specific CD8⁺ T cells with an activated phenotype, likely reflecting IL-6-mediated hyperinflammation. We propose that FGL-1 functions as an IL-6-induced acute-phase protein with immunomodulatory effects, potentially limiting effector T cell-driven hyperinflammation and T-cell activation via LAG-3 engagement.

Wang et al.^22^ demonstrated that FGL-1 mediates LAG-3-dependent T-cell suppression both in vitro and in vivo, identifying FGL-1 as a potential key immune checkpoint ligand. Additionally, Yang et al.^42^ found that the addition of FGL-1 upregulated LAG-3 expression on CD8⁺ tissue-resident memory T cells in vitro, suggesting that FGL-1 may impair the cytotoxic function of CD8⁺ T_RM_ cells through LAG-3 engagement. This finding implies a potential feedback mechanism in which FGL-1 not only binds to LAG-3 but also promotes its expression, further reinforcing T-cell suppression. Furthermore, Chen et al.^53^ demonstrated that the addition of FGL-1-Fc protein in vitro suppressed the response of regulatory T cells, which highly expressed LAG-3, thereby further supporting the immunomodulatory role of FGL-1 through its binding to LAG-3.

Notably, the study by Maruhashi et al.^30^ indicates that stable peptide-MHC class II, rather than FGL-1, is the dominant mediator of LAG-3 inhibitory activity in vitro and in vivo; nevertheless, accumulating evidence indicated that the FGL-1/LAG-3 axis plays a key immunomodulatory role. This is evidenced by its capacity to suppress T-cell responses, upregulate LAG-3 surface expression, and by the therapeutic efficacy of anti-FGL-1 antibodies, which is abrogated in LAG-3-deficient models, suggesting context-dependent and potentially complementary mechanisms of immune regulation^22,42,44^.

In the context of COVID-19, the upregulation of the LAG-3/FGL-1 pathway is likely driven by a SARS-CoV-2-specific immune response; it may also be observed in other chronic viral infections associated with immune cell dysfunction. Considering its role in cancer immune evasion and immunosuppression in the tumor microenvironment^22,24^, our findings may suggest that elevated FGL-1 levels contribute to this immune dysfunction by engaging LAG-3 on activated CD8^+^ T cells, potentially dampening effective antiviral responses.

While in the contexts mentioned above the FGL-1/LAG-3 pathway might rather be dysregulated or exploited by the tumor microenvironment, FGL-1 upregulation during acute infections may also represent a physiological negative-feedback mechanism aimed at limiting excessive immune activation. Through interaction with LAG-3 on effector cells, FGL-1 could contribute to the prevention of immunopathology and tissue damage by broadly dampening inflammatory responses, although this may simultaneously impair virus-specific immunity. Overall, the data we and others provided on FGL-1 in different contexts suggest a common mechanism of immunomodulation during inflammation and infection.

A key strength of this study is the availability of detailed clinical data, combined with matched plasma and PBMC samples from a relatively large and well-characterized cohort of unvaccinated patients with COVID-19, collected early in the pandemic. This unique setting allowed us to assess the immune response to SARS-CoV-2 infection without the influence of prior immunity induced by vaccination or emerging viral variants. The longitudinal nature of sampling in a patient with severe COVID-19 disease further enabled the assessment of FGL-1 dynamics in relation to disease progression and clinical outcome.

However, our study also has limitations. The data were collected before the emergence of SARS-CoV-2 variants such as Omicron, which are associated with altered clinical courses and immune responses. Future studies should aim to replicate and extend our findings in vaccinated individuals, as well as in the context of ongoing viral evolution. Importantly, liver-derived FGL-1 may be particularly relevant in specific patient subgroups, such as those with pre-existing liver conditions like liver cirrhosis and HCC. Follow-up studies should investigate FGL-1 levels and immune signatures in patients with HCC, liver cirrhosis, and other hepatic comorbidities infected with SARS-CoV-2 or other pathogens^53,54^. This would help to further study the interplay between liver inflammation, FGL-1 secretion, and systemic immune modulation.

We acknowledge that the analysis of SARS-CoV-2-specific CD8⁺ T cells is based on a limited sample size, which should be considered when interpreting these results. Further in-depth immunological analyzes should examine LAG-3 expression across distinct lymphocyte populations beyond CD8^+^ T cells. Regulatory T-cell subsets, particularly type 1 regulatory T cells (Tr1), which show a canonical co-expression of LAG-3 and CD49b and are involved in immunosuppression, may represent important targets for functional interactions between FGL-1 and LAG-3^55^. Given its broad-acting effects, FGL-1 should also be evaluated for its immunomodulatory roles in chronic inflammatory and autoimmune diseases, where aberrant checkpoint signaling may contribute to pathogenesis.

In summary, plasma FGL-1 levels were elevated in patients with COVID-19 and correlated with markers of inflammation and immune cell activation. Furthermore, its receptor, LAG-3, was significantly upregulated in both bulk and SARS-CoV-2-specific CD8^+^ T cells. These findings highlight FGL-1’s potential not only as an acute-phase protein in systemic inflammation but also as a functional mediator of immune suppression via the LAG-3 axis. Future studies are needed to understand the functional relevance of the FGL-1/LAG-3 axis in modulating immune activation and T cell responses and its role in COVID-19 and other diseases where immune modulation is critical, similar to its emerging role in cancer immunotherapy. Our findings are based on observational, retrospective data; therefore, further investigation is required to elucidate the functional mechanisms underlying the FGL-1/LAG-3 axis in COVID-19. In particular, understanding the precise cellular sources and downstream effects of FGL-1 in COVID-19 could further clarify its dual role in inflammation and immune regulation.

## Supplementary information

Transparent Peer Review file

Supplemental Material

Description of Additional Supplementary Files

Supplementary Data 1

## Data Availability

Data storage is performed by the University Hospital Hamburg-Eppendorf, Hamburg, Germany. Data and corresponding source data are available in the corresponding tabs of the Supplementary Data file and can be shared after confirming that data will be used within the scope of the originally provided informed consent.
