# Supplementary material for: FGL-1 plasma levels correlate with markers of inflammation and severe disease during SARS-CoV-2 infection
Source: Commun Med (Lond). 2026 Jul 31;6:439. doi: 10.1038/s43856-026-01775-4 (PMC13469107; doi:10.1038/s43856-026-01775-4)
Supplement: Supplementary file 1 [file 43856_2026_1775_MOESM1_ESM.pdf]

nature portfolio

Peer Review File

FGL-1 plasma levels correlate with markers of inflammation and severe disease during SARS-CoV-2 infection.

Corresponding Author: Professor Julian Schulze zur Wiesch

This file contains all reviewer reports in order by version, followed by all author rebuttals in order by version.

Version 0:

Reviewer comments:

Reviewer #1

(Remarks to the Author)
This manuscript investigates the role of FGL-1, a liver-derived immunomodulatory protein and ligand for LAG-3, in the pathogenesis of acute COVID-19. The authors show that plasma FGL-1 levels are significantly elevated in patients with COVID-19 and correlate with IL-6, CRP, and disease severity. Furthermore, the study identifies increased expression of LAG-3 on bulk and SARS-CoV-2-specific $CD8^+$ T cells, which is associated with co-expression of activation markers. The authors propose that FGL-1 functions as an IL-6-induced acute-phase protein that may regulate T-cell activation and immune responses through engagement of LAG-3, potentially contributing to immune modulation during acute infection. This is a descriptive study in nature without any novel mechanistic insight. The lack of functional validation and statistical depth, reduce the strength of the conclusions.

The discussion, abstract, and parts of the results section contain overstatements, with claims based on weak correlations. For example, the statement, “Understanding the functional relevance of this axis on immune cells could provide novel insights into therapeutic targets for managing severe COVID-19 and other diseases where immune modulation is critical,” is not supported by the data. This study does not provide mechanistic evidence or results with therapeutic implications. Additionally, in the abstract, the authors claim that FGL-1, via Gal-3, may limit excessive inflammation; however, no evidence is presented to support this conclusion.

Major Concerns
A) Absence of functional validation
The central conclusion that FGL-1 contributes to T-cell modulation via LAG-3 is primarily based on correlative data. The study lacks functional experiments demonstrating direct effects of FGL-1 on T-cell responses, such as cytokine production, proliferation, or cytotoxicity assays following FGL-1 stimulation or blockade. Without such validation, the mechanistic conclusions remain speculative.
B) Limited statistical and multivariate analyses
The observed correlations between FGL-1 levels and disease severity or inflammatory markers may be confounded by additional clinical variables such as age, comorbidities, or overall inflammatory burden. Multivariate analyses would significantly strengthen the biomarker claims and improve the robustness of the conclusions.
C) Interpretation of T cell exhaustion
The classification of T cells as functionally exhausted is largely based on phenotypic marker expression. The authors use the terms exhausted and activated T cells interchangeably. Basic immunological principles link T cell exhaustion to chronic stimulation or persistent antigen exposure. However, COVID-19 is an acute disease, and from an immunological standpoint, T cell exhaustion would not be expected.
Additionally, activated T cells upregulate co-inhibitory receptors as part of normal immune self-regulation and homeostasis. Therefore, the authors should avoid using exhaustion terminology unless it is supported by functional assays. The authors may consider discussing this study, which demonstrated upregulation of multiple co-inhibitory receptors, including LAG-3, in acute COVID-19, while also showing, through functional assays, that both bulk and antigen-specific T cells were activated but not exhausted (PMID: 34312258).
Please exclude references that classify T cells as exhausted in acute COVID-19 based solely on co-inhibitory receptor expression in the absence of functional assays.

D) Small sample sizes in mechanistic subgroups

While the overall cohort size is acceptable, key mechanistic analyses, particularly antigen-specific T-cell studies, involve relatively small sample numbers. This limits statistical power and generalizability and should be acknowledged more prominently.
E) Incomplete mechanistic insight into FGL-1 source and regulation
Although FGL-1 is described as primarily liver-derived, the manuscript does not directly investigate the cellular sources of FGL-1 or the relationship between hepatic injury and FGL-1 production. Additional discussion or experimental evidence addressing this aspect would improve mechanistic clarity.
F) It is unclear why the authors focused only on IL-6 and CRP. Acute COVID-19 is associated with a cytokine storm, and multiple cytokines and chemokines are elevated depending on disease severity, including TNF-α, IP-10, IL-15, MCP-1, and SAA (PMID: 33947753). Therefore, a clear justification for restricting the analysis to IL-6 and CRP is needed.
Fig 1.
R^2 values are low, particularly for the IL-6/FGL-1 correlation ($R^2 = 0.23$), indicating that this association explains only about 20–25% of the data. Similarly, the correlation between CRP and FGL-1 is modest ($R^2 = 0.43$).
Fig. 2
This observation appears to reflect the expected resolution of disease severity, whereby most inflammatory biomarkers, not limited to FGL-1 and CRP, decline within two weeks after the acute phase. Consequently, this figure does not offer novel mechanistic or clinical insight.
Fig. 3
Representative flow cytometry plots for activation and co-inhibitory receptors should be included in this figure. The reported co-expression of LAG-3 with CD38 and HLA-DR primarily indicates T-cell activation rather than exhaustion. Moreover, a reduction in $CD73^+$ $CD8^+$ T cells in acute COVID-19 patients has already been documented (PMID: 34312258). In the absence of representative gating and expression plots, it is not possible to draw firm conclusions from these data.
Fig. 4
A major caveat of this analysis is the absence of an appropriate negative control for tetramer staining. The specificity of the tetramer signal must be validated. The most appropriate approach would be to use PBMCs or $CD8^+$ T cells from HLA-A*02–negative individuals to confirm the absence of the reported tetramer-positive population. Without such a negative control, the identity and fate of $tetramer^+$ T cells cannot be conclusively determined.
Other comments
1. The inclusion of vaccinated individuals in the healthy control group may introduce variability and should be clarified or statistically addressed.
2. Some sections of the discussion are repetitive, particularly regarding IL-6-mediated FGL-1 regulation.
3. Much of the supplemental correlational data shows very low R^2 values (e.g., $R^2 = 0.10$–0.18 for LDL versus lymphocyte counts). Such weak correlations are likely incidental and lack biological significance, as more than 80–85% of the variability in the data is not explained by these associations.
4. SARS-CoV-2 isolates exhibit differential immunological outcomes, particularly in cytokine/chemokine profiles and co-inhibitory receptors, including soluble forms such as TIM-3, TIGIT, PD-1, and LAG-3. Interestingly, Omicron appears to induce more pronounced LAG-3 shedding compared to Delta and Wuhan strains. If elevated LAG-3 is associated with disease severity, how might the authors explain this observation (PMCID: PMC10581158). Additionally, the viral isolate/strain should be clearly mentioned in the methods section.
5. The manuscript would benefit from clearer differentiation between hypothesis-generating observations and experimentally validated conclusions.
6. The longitudinal analysis relies heavily on pooled data and a single extended case study, which limits interpretation of temporal dynamics.

Reviewer #2

(Remarks to the Author)
Authors have investigated the expression of circulating FGL-1 in patients with COVID-19 and have stablished a correlation with other well-known inflammatory markers. Furthermore, the study characterizes LAG-3+ immune cells in SARS-CoV-2-infected individuals and hypothesizes about the possible role of FGL-1 through the interaction with LAG-3 in modulating the immune response during acute COVID-19.
The study is well designed, well written and is easy to follow. This is an observational and mainly retrospective study. Although authors fail to demonstrate that FGL-1 is the main ligand responsible of LAG-3-driven T cell exhaustion during infection, this piece of work unveils some interesting observations that may lead to further research on immune cell dysfunction in severe COVID-19.
Authors should address the following points:
1. Line 100: authors describe a HD cohort consisting of 20 uninfected individuals. However, for T cell characterization studies, only 4 of them are shown in the graphs. The number of HD included in these studies should be increased if possible, to avoid a potential bias. Otherwise, it should be stated in methods why only 4 patients were included in these analyses and if these were the unvaccinated ones. Is it because vaccination may alter CD8 LAG-3+ cells? Please clarify this point.
2. Line 154: please specify whether normality tests to choose the adequate parametric or non-parametric statistical test were employed or if only non-parametric methods were used due to restricted sample size.
3. Figure 1B: a trend test across ordered groups (e.g. Cuzick) would be informative to assess statistical significance with increased severity.
4. Figure 1: in some cases, saturation of ELISA test may preclude from assessing differences between groups. Were titration curves performed to select the best dilution? If so, please include them. Also, include in Fig.1 legend the dilutions used for panels A and B.

5. Figure 2: it would be interesting to include a ROC curve showing a comparison of the severity classification power between FGL-1, IL-6 and CRP. Line 486: it is not clear to me why spearman test was used in this case (as stated in the figure legend) and not a test for time series.
6. Figure 3C: the plots seem to be paired. Please clarify if paired or unpaired analyses were used for each panel. Matched lines between dots would improve interpretation.
7. Figure 4: I wonder whether it would be more informative to compare Cov-specific cells with non-specific (non-tetramer+) instead of bulk CD8. Fig. 4C, please include percentage of each gate in flow cytometry plots. In addition, splitting analysis by severity would help interpretation and may be included as a supplementary figure.
8. As a general comment, I wonder if authors have tried to assess levels of certain proteins downstream of LAG-3 (ZAP-70, STAT-1) in critical vs mild COVID-19 to functionally characterize this possible FGL-1-driven immunosuppressive action.
9. Table 1: please add the clinical characteristics of the healthy donors included in the study.
10. Line 138: a representative gating of the LAG3+ cells selection with the corresponding percentages should be incorporated to supplementary material.
11. Supplementary Figure 3: please add the p-value of the comparison between COVID-19 and chronic HCV as stated in line 187 of the manuscript.
12. Supplementary Figure 6: a trend test across ordered groups (e.g. Cuzick) could also be used to determine if rising severity associates with less CD8+ T cells.
13. Supplementary Figure 7: panel letters do not match with panel figure.
14. Supplementary Figure 10: authors should include in the legend that Tet+ stands for tetramer positive cells. The correlation coefficient (R) should be added.
15. Line 376: authors mention a study that functionally associates FGL-1 with LAG-3. However, there are other studies that highlight that FGL-1 may be dispensable for LAG-3-mediated immunosuppression (see Maruhashi et al., 2022 https://pubmed.ncbi.nlm.nih.gov/35413245/). Thus, authors should be cautious when discussing the FGL-1/LAG-3 axis and its role in COVID-19 physiopathology, since evidence seems contradictory. This is important for the conclusions of the study and it should be discussed. Moreover, authors should include in the limitations section that their study is observational and that new studies aimed to investigating the functional mechanisms behind FGL-1/LAG-3 axis are needed.

Version 1:

Reviewer comments:

Reviewer #1

(Remarks to the Author)
The manuscript has benefited from additional work and revisions. My comments have been addressed.

Reviewer #2

(Remarks to the Author)
The authors have thoroughly addressed all the major and minor concerns raised in the initial review. The revised manuscript shows significant improvement in clarity, figure presentation, and data integration.

Open Access This Peer Review File is licensed under a Creative Commons Attribution 4.0 International License, which permits use, sharing, adaptation, distribution and reproduction in any medium or format, as long as you give appropriate credit to the original author(s) and the source, provide a link to the Creative Commons license, and indicate if changes were made.
In cases where reviewers are anonymous, credit should be given to 'Anonymous Referee' and the source.
The images or other third party material in this Peer Review File are included in the article's Creative Commons license, unless indicated otherwise in a credit line to the material. If material is not included in the article's Creative Commons license and your intended use is not permitted by statutory regulation or exceeds the permitted use, you will need to obtain permission directly from the copyright holder.
To view a copy of this license, visit https://creativecommons.org/licenses/by/4.0/
