# Supplementary material for: FGL-1 plasma levels correlate with markers of inflammation and severe disease during SARS-CoV-2 infection
Source: Commun Med (Lond). 2026 Jul 31;6:439. doi: 10.1038/s43856-026-01775-4 (PMC13469107; doi:10.1038/s43856-026-01775-4)
Supplement: Supplementary file 2 [file 43856_2026_1775_MOESM2_ESM.pdf]

Supplementary Material

FGL-1 plasma levels correlate with markers of inflammation and severe disease during SARS-CoV-2 infection.

Sophia Cichutek[1], Leon Cords[2], Kilian Keetz[2], Marissa Herrmann[2,3], Liz J. Lam[2], Maria Mader[2,3], Robin Woost[2,3], Stefan Schmiedel[2], Ansgar W. Lohse[2,3], Carsten Bokemeyer[1], Christoph Schultheiß[4,5], Marylyn M. Addo[2,3,6], Walter Fiedler[1], Mascha Binder[4,5,6], Julian Schulze zur Wiesch[2,3]

[1] Department of Oncology, Hematology and Bone Marrow Transplantation with Section Pneumonology, University Medical Center Hamburg-Eppendorf, Hamburg, Germany

[2] I. Department of Medicine, University Medical Center Hamburg-Eppendorf, Hamburg, Germany.

[3] German Center for Infection Research (DZIF), Partner Site Hamburg-Lübeck-Borstel-Riems, Hamburg, Germany.

[4] Division of Medical Oncology, University Hospital Basel, Basel, Switzerland.

[5] Laboratory of Translational Immuno-Oncology, Department of Biomedicine, University, and University Hospital Basel, Basel, Switzerland.

[6] Institute for Infection Research and Vaccine Development (IIRVD), University Medical Center Hamburg-Eppendorf, Hamburg, Germany

* Corresponding author:
Prof. Dr. med. Julian Schulze zur Wiesch, MD
I. Department of Medicine
University Medical Center Hamburg-Eppendorf
20246 Hamburg
Germany
Telephone number: +49 40 7410 52831
E-mail address: j.schulze-zur-wiesch@uke.de

Supplementary Tables

	Healthy donors<br>n = 33
Age in years, mean (range)	32 (21-61)
Sex at birth, n (%)	
female	13 (39.4%)
male	20 (60.6%)
Comorbidities, n (%)	
none	33 (100%)
COVID-19 vaccination, n (%)	
no	12 (36.4%)
yes	21 (63.6%)
SARS-CoV-2 infection, n (%)	
no	12 (36.4%)
resolved	21 (63.6%)

Supplementary Table 1. Characteristics of healthy donors in the clinical cohort. Values are presented as absolute numbers, percentages, or means with ranges. Laboratory results were not available.

	Definition	Symptoms
Mild	No pneumonia	Mild symptoms (e.g., fever, cough, or change in taste or smell, no dyspnea)
Moderate	Pneumonia	No symptoms of severe pneumonia
Severe	Severe pneumonia	Defined by bilateral infiltrations in the lungs and/or respiratory rate >30/min and/or severe dyspnea and/or peripheral oxygen saturation <90%
Critical	ARDS	ARDS criteria and/or septic disease with multi organ failure

Supplementary Table 2. Disease severity classification of COVID-19. Disease severity was determined according to the German STAKOB guidelines [1] based on the clinical presentation of the patients.

Panel 1 – Immunophenotypic characterization						
Laser	Fluorophore	Antigen	Clone	Manufacturer	Cat. No.	Dilution
UV 355 nm	BUV395	CD16	3G8	BD	563785	1:40
	BUV496	CD3	SK7	BD	569786	1:40
	BUV563	CD45RA	HI100	BD	612926	1:500
	BUV615	CD38	HIT2	BD	751138	1:100
	BUV661	CD73	AD2	BD	750060	1:200
	BUV737	CD19	SJ25C1	BD	612756	1:200
	BUV805	CD8	SK1	BD	612889	1:67
Violet 405 nm	BV421	LAG-3	11C3C65	BioLegend	369314	1:40
	BV510	CD4	SK3	BD	562970	1:200
	BV605	TIGIT	A15153G	BioLegend	372712	1:67
	BV650	CD14	M5E2	BioLegend	301836	1:67
	BV711	CD49d	9F10	BioLegend	304332	1:100
	BV785	CD27	O323	BioLegend	302832	1:100
Blue 488 nm	FITC	CD56	5.1H11	BioLegend	362546	1:100
	PerCP/Cy5.5	IgD	IA6-2	BioLegend	348208	1:67
Y/G 561 nm	PE	CD25	M-A251	BioLegend	356104	1:200
	PE/Dazzle 594	PD-1	EH12.2H7	BioLegend	329940	1:200
	PE/Cy7	CD39	A1	BioLegend	328212	1:200
Red 637 nm	APC	CD127	A019D5	BioLegend	351316	1:200
	Alexa Fluor 700	HLA-DR	L243	BioLegend	307626	1:67
	LIVE/DEAD Fixable Near-IR Dead Cell Stain Kit			Invitrogen	L10119	1:200
Panel 2 – SARS-CoV-2-specific CD8^{+} T cell phenotyping						
Laser	Fluorophore	Antigen	Clone	Manufacturer	Cat. No.	Dilution
UV 355 nm	BUV395	CD39	A1	BD	567507	1:100
	BUV496	CD3	SK7	BD	569786	1:40
	BUV563	CCR7	3D12	BD	741317	1:50
	BUV615	CD38	HIT2	BD	751138	1:133
	BUV737	CD73	AD2	BD	612812	1:67
	BUV805	CD8	HIT8a	BD	742030	1:67
Violet 405 nm	BV421	LAG-3	11C3C65	BioLegend	369314	1:40
	BV510	CD57	QA17A04	BioLegend	393314	1:133
	BV605	CD244	C1.7	BioLegend	329536	1:67
	BV650	CD4	RPA-T4	BioLegend	300536	1:100
	BV711	TIGIT	A15153G	BioLegend	372742	1:67
	BV785	CD127	A019D5	BioLegend	351330	1:40
Blue 488 nm	FITC	KLRG1	SA231A2	BioLegend	367714	1:100
	PerCP/Cy5.5	HLA-DR	L243	BioLegend	307630	1:67
Y/G 561 nm	PE	BTLA	J168-540	BD	558485	1:50
	PE/Dazzle 594	TIM-3	F38-2E2	BioLegend	345034	1:50
	PE/Cy7	PD-1	EH12.2H7	BioLegend	329918	1:67
Red 637 nm	APC	A*02:01-YLQPRTFLL	—	NIH		1:200
	Alexa Fluor 700	CD45RA	HI100	BioLegend	304120	1:125
	APC-Cy7	CD14	63D3	BioLegend	367108	1:200
	APC-Cy7	CD19	HIB19	BioLegend	302218	1:200
	LIVE/DEAD Fixable Near-IR Dead Cell Stain Kit			Invitrogen	L10119	1:200

Supplementary Table 3. Flow cytometry panels. The flow cytometry panels in this study were used to characterize LAG-3 expression of immune cell subsets (Panel 1) and SARS-CoV-2-specific CD8^{+} T cells (Panel 2) in patients with COVID-19 and healthy donors.

	regression coefficient (β)	95% CI	p-value	p-value summary
age	-1.309	-24.01 to 21.39	0.9081	ns
sex (male)	-69.09	-717.6 to 579.4	0.8311	ns
disease severity (critical)	809.8	-606.4 to 2226	0.2557	ns
disease severity (moderate)	31.67	-1188 to 1251	0.9585	ns
disease severity (severe)	266.9	-1051 to 1585	0.6855	ns
comorbidities (any)	-394.1	-1394 to 605.7	0.4316	ns
lymphocyte count	-141.2	-392.2 to 109.8	0.2634	ns
ALT	-4.660	-14.35 to 5.028	0.3380	ns
CRP	10.03	3.941 to 16.12	0.0018	**
LDH	0.9173	-2.059 to 3.893	0.5381	ns
IL-6	0.5263	-4.950 to 6.003	0.8475	ns
ferritin	0.2212	-0.2612 to 0.7037	0.3608	ns

Supplementary Table 4. Multiple linear regression analysis of factors associated with FGL-1 levels. Multiple linear regression analysis was performed in GraphPad Prims v10.6.1 to examine the relationship between FGL-1 levels and the predictors age, sex, disease severity, comorbidities (any), lymphocyte count, IL-6, CRP, LDH, ALT and ferritin levels. Multicollinearity was examined using variance inflation factors. Results are presented as regression coefficients (β) with 95% confidence intervals (CI) and corresponding p-values (≥0.05 (ns), <0.05 (*), <0.01 (**), <0.001 (***)). ALT, alanine transaminase; CRP, C-reactive protein; LDH, lactate dehydrogenase; IL-6, Interleukin-6.

A – COVID-19 patients							
Donor	Age in years	Sex (F, female; M, male)	COVID-19 severity	Days since diagnosis	FGL-1 [ng/ml]	CRP [mg/l]	IL-6 [ng/l]
COV-01	62	F	mild	1	196.5	3	6.3
COV-02	27	F	mild	8	1101.2	3	<2.7
COV-03	39	M	mild	21	2945.8	46	4.7
COV-04	38	F	mild	1	1474.0	6	n.a.
COV-05	68	F	moderate	10	2420.3	26	71.1
COV-06	76	F	moderate	1	807.3	7	14.8
COV-07	77	M	moderate	8	985.2	22	9.4
COV-08	67	M	moderate	17	166.5	4	1.8
COV-09	73	F	moderate	4	789.2	50	11
COV-10	36	F	moderate	3	1239.5	16	7.6
COV-11	53	F	moderate	8	1828.1	26	26.6
COV-12	51	F	moderate	5	4199.4	125	45
COV-13	59	M	moderate	7	2009.6	72	49.8
COV-14	56	F	moderate	7	1607.0	38	19
COV-15	75	M	severe	36	3751.0	41	29.2
COV-16	74	M	severe	3	3615.6	95	17.4
COV-17	65	M	severe	6	2949.9	92	49.4
COV-18	77	M	severe	3	3847.0	36	19
COV-19	36	M	severe	9	139.7	4	2.7
COV-20	74	M	severe	5	4138.2	129	69.7
COV-21	69	M	critical	35	2501.1	63	12.8
COV-22	35	F	critical	50	1705.1	4	1.7
COV-23	76	F	critical	4	3752.5	207	230
COV-24	63	M	critical	24	2584.6	76	58
COV-25	74	F	critical	18	3021.6	70	28
COV-26	63	M	critical	24	2584.6	76	58
COV-27	82	M	critical	22	1566.5	44	7.3

B – Healthy Donors						
Donor	Age in years	Sex (F, female; M, male)	Date of sample collection	COVID-19 vaccination	Resolved SARS-CoV-2 infection	comorbidities
HD-01	27	M	08.04.21	yes	yes	none
HD-02	28	M	17.09.21	yes	yes	none
HD-03	47	M	26.01.22	yes	yes	none
HD-04	51	F	16.08.21	yes	yes	none
HD-05	47	M	08.10.21	yes	yes	none
HD-06	28	M	17.09.21	yes	yes	none
HD-07	26	M	24.07.25	yes	yes	none
HD-08	25	M	04.10.24	yes	yes	none
HD-09	28	F	30.09.25	yes	yes	none
HD-10	27	M	31.03.25	yes	yes	none
HD-11	23	M	07.12.23	yes	yes	none
HD-12	25	F	08.12.25	yes	yes	none
HD-13	26	M	19.08.25	yes	yes	none
HD-14	32	F	03.11.24	yes	yes	none

Supplementary Table 5. Immunological study cohort of (**A**) COVID-19 patients (n = 27) and (**B**) healthy donors (n = 14). Time since diagnosis refers to the time passed since the first positive PCR swab. Laboratory results are derived from the clinical database from the day of the sampling ±2 days. Abbreviations: FGL-1, Fibrinogen-like protein 1; CRP, C-reactive protein; IL-6, Interleukin-6. Reference values of laboratory results: IL-6 <7 ng/l; CRP <5 mg/l.

Donor	Age in years	Sex (F, female; M, male)	COVID-19 severity	Days since diagnosis	FGL-1 [ng/ml]	CRP [mg/l]	IL-6 [ng/l]	HLA class I molecules
TET-01	74	M	severe	5	4138.2	129	69.7	HLA-A*02:01
TET-02	63	M	critical	24	2584.60	76	58	HLA-A*02:01
TET-03	36	F	moderate	3	1239.51	16	7.6	HLA-A*02:01
TET-04	53	F	moderate	8	1828.12	26	26.6	HLA-A*02:01
TET-05	51	F	moderate	5	4199.41	125	45	HLA-A*02:01
TET-06	59	M	moderate	7	2009.62	72	49.8	HLA-A*02:01
TET-07	56	F	moderate	7	1607.05	38	19	HLA-A*02:01
TET-08	75	M	severe	36	3750.97	41	29.2	HLA-A*02:01
TET-09	74	M	severe	3	3615.63	95	17.4	HLA-A*02:01
TET-10	65	M	severe	6	2949.94	92	49.4	HLA-A*02:01
TET-11	35	F	critical	50	1705.10	4	1.7	HLA-A*02:01

Supplementary Table 6. Immunological study cohort for virus-specific $CD8^+$ T cell analysis. Time since diagnosis refers to the time passed since the first positive PCR swab. Laboratory results are derived from the clinical database from the day of the sampling ±2 days. Abbreviations: FGL-1, Fibrinogen-like protein 1; CRP, C-reactive protein; IL-6, Interleukin-6. Reference values of laboratory results: IL-6 <7 ng/l; CRP <5 mg/l.

Supplementary Figures

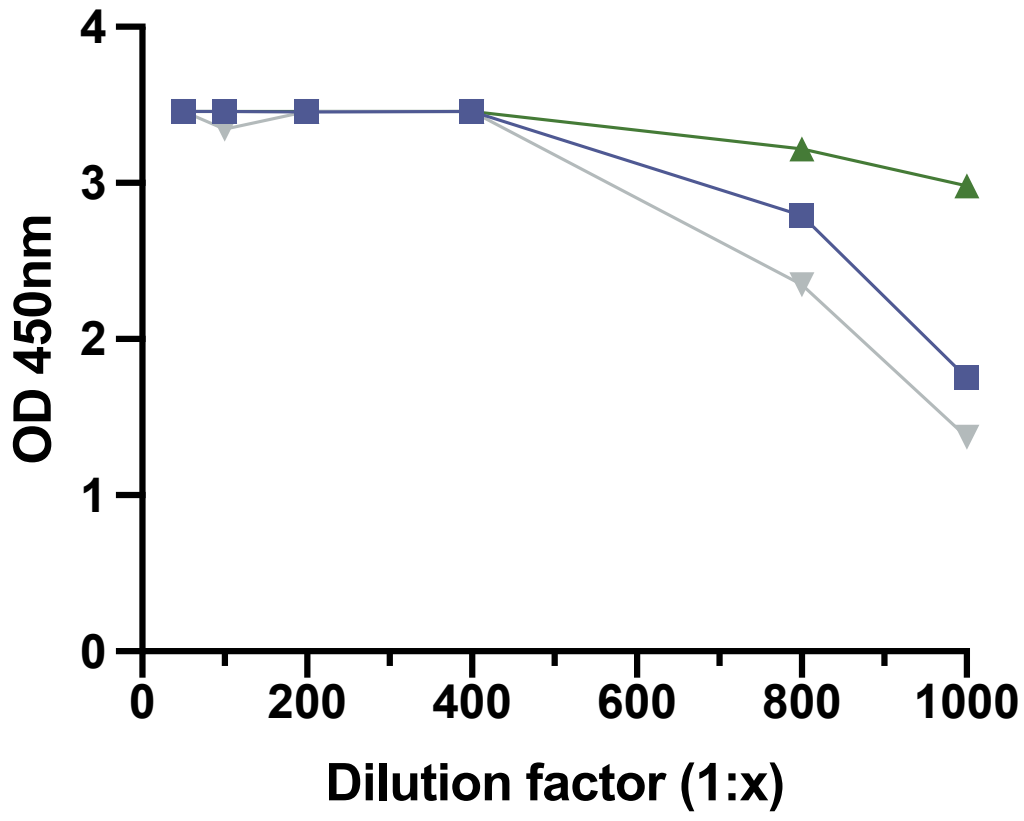

Supplementary Figure 1. Titration curve of three representative samples of COVID-19 patients showing the optical density (OD) measured at 450 nm at different dilution factors to determine the optimal dilution of samples for the FGL-1 ELISA measurements. A final dilution of 1:1000 was used for the experiments.

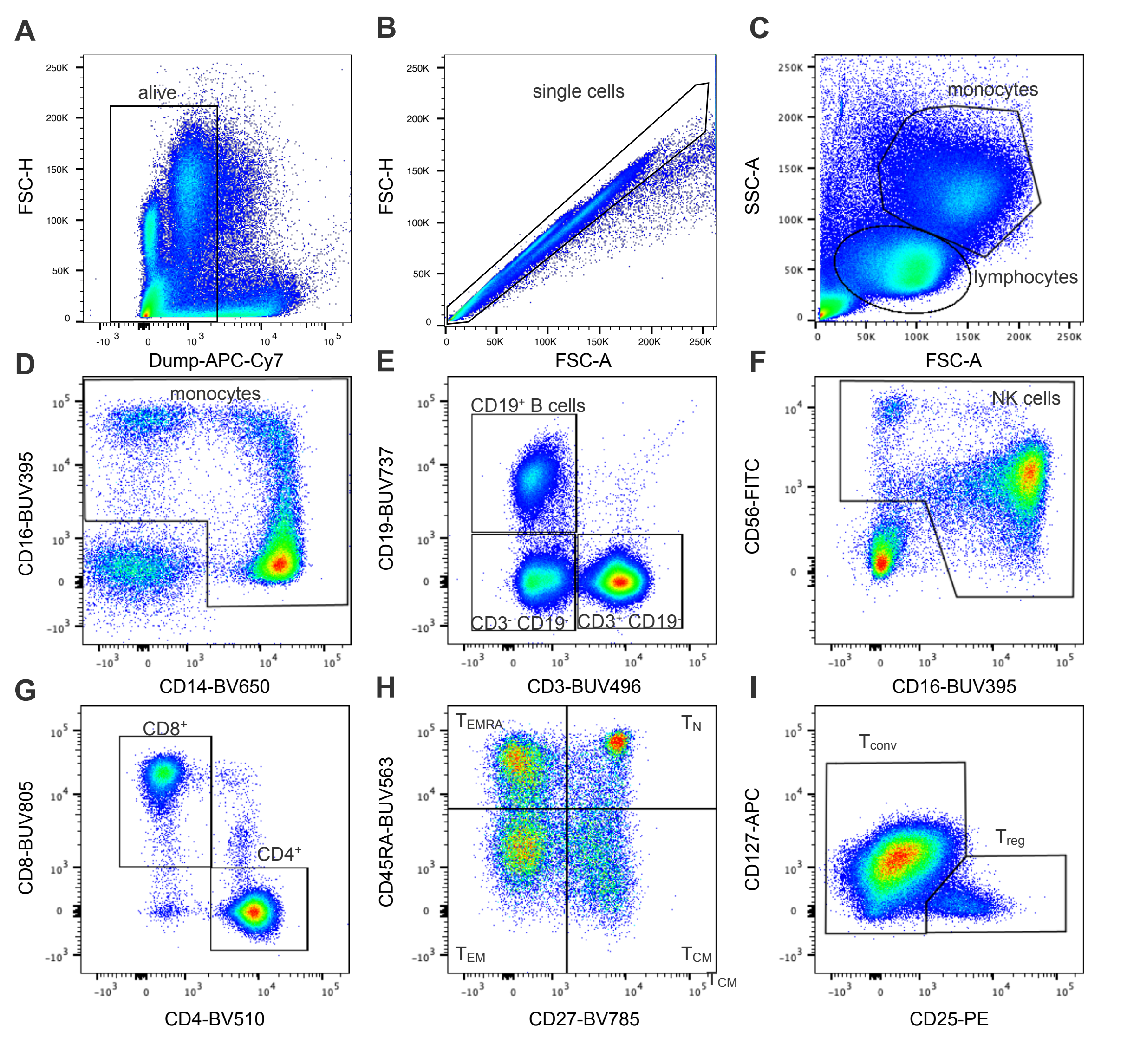

Supplementary Figure 2. Representative dot plots showing the gating strategy of immune cell phenotyping. (**A**) Only live cells were included. (**B**) Doublets were excluded by a forward scatter height (FSC-H) and forward scatter area (FSC-A) gate. (**C**) Initial gating strategy to identify lymphocyte and monocyte populations. (**D**) Monocytes were identified from the monocyte gate shown in (A) as CD14^{++}/CD16^{-}, CD14^{++}/CD16^{+} and CD14^{+}/CD16^{+} cells. (**E**) B cells were identified from the lymphocyte gate shown in (A) as CD3^{-}/CD19^{+} cells. (**F**) NK cells were identified from the CD19^{-}/CD3^{-} lymphocyte population as CD56^{+}/CD16$^{+/-}$ cells. (**G**) T cells were identified from the CD19^{-}/CD3^{+} lymphocyte population as CD4^{+} and CD8^{+} T cells. (**H**) CD8^{+} T-cell subsets were classified as naïve (T_N), central memory (T_{CM}), effector memory (T_{EM}), and terminally differentiated effector memory (T_{EMRA}) cells. (**I**) CD4^{+} T-cell subsets were classified as regulatory T cells (T_{reg}) and conventional T cells (T_{conv}). Antibodies used are provided in **Supplementary Table 3**.

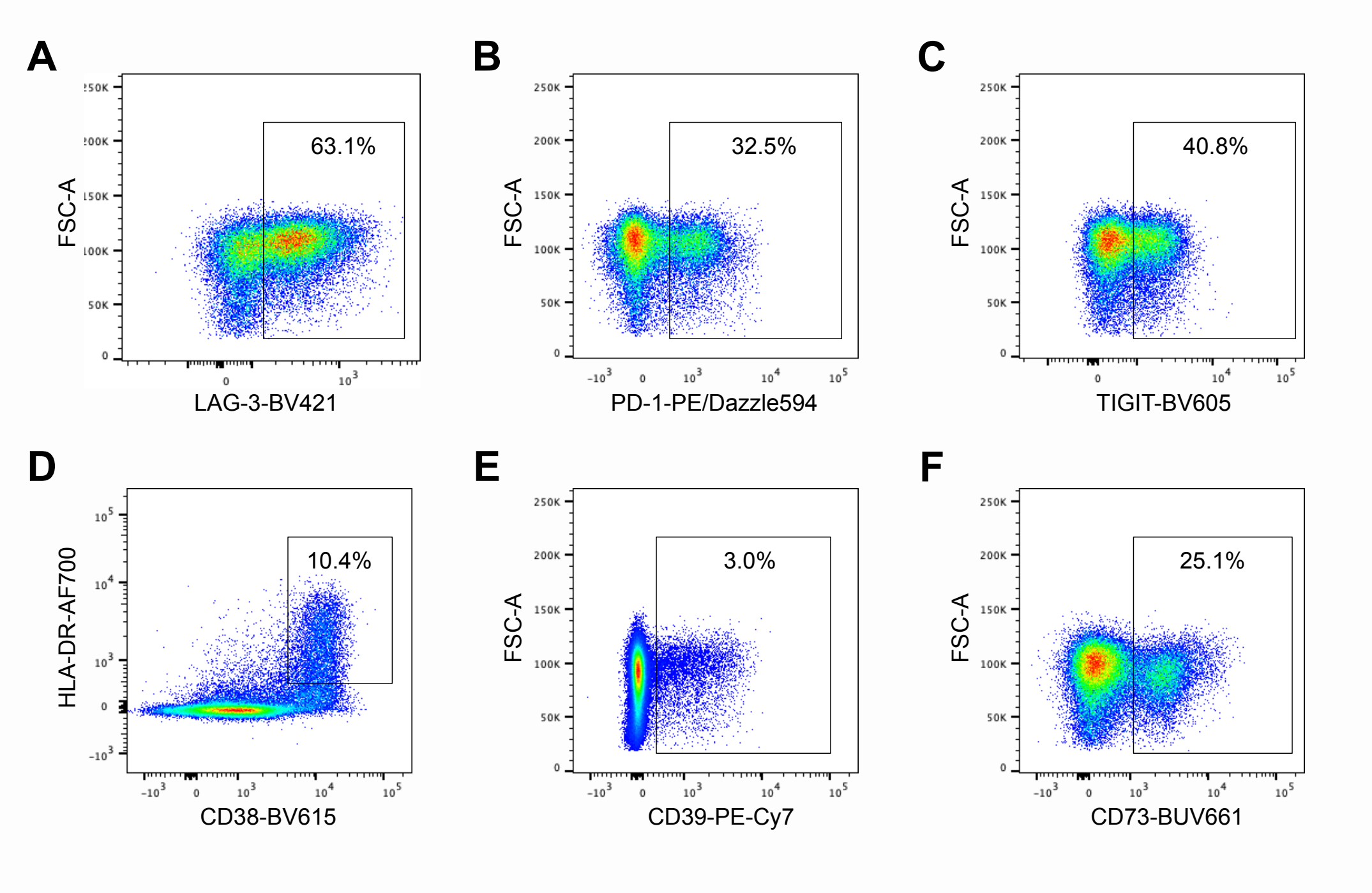

Supplementary Figure 3. Representative dot plots gated on CD8⁺ T cells showing the gating of the (**A**) LAG-3⁺, (**B**) PD-1⁺, (**C**) TIGIT⁺, (**D**) CD38⁺/HLA-DR⁺, (**E**) CD39⁺ and CD73⁺ population in patients with COVID-19.

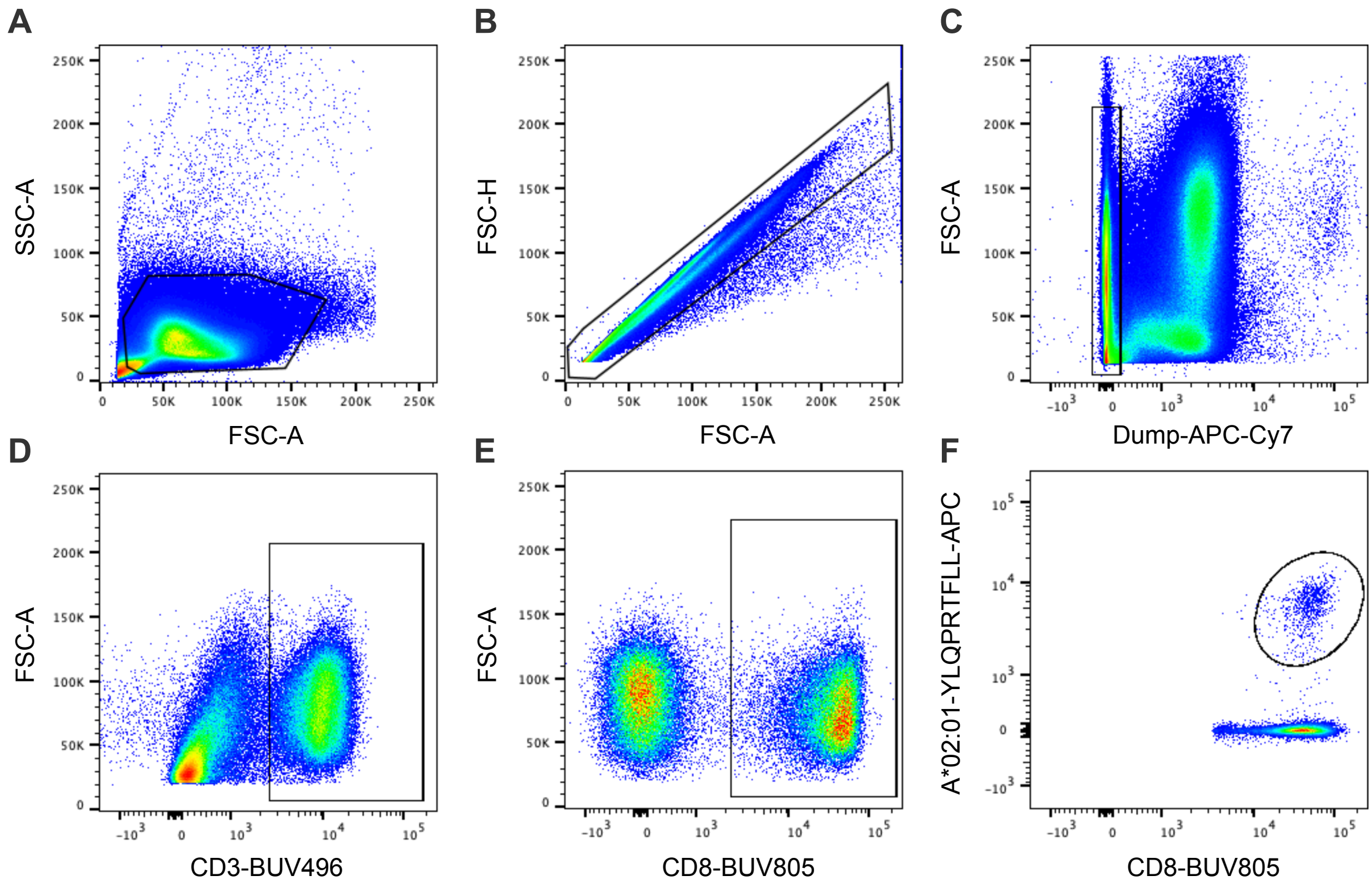

Supplementary Figure 4. Representative dot plots showing the gating strategy of PBMCs. (**A**) Lymphocytes were first identified by a low forward scatter (FSC) and low side scatter (SSC) gate. (**B**) Doublets were excluded by a forward scatter height (FSC-H) and forward scatter area (FSC-A) gate. (**C**) Only live cells were included. (**D**) Only $CD3^+$ cells were included. (**E**) $CD8^+$ T cells were identified and (**F**) tetramer-positive cells were gated on.

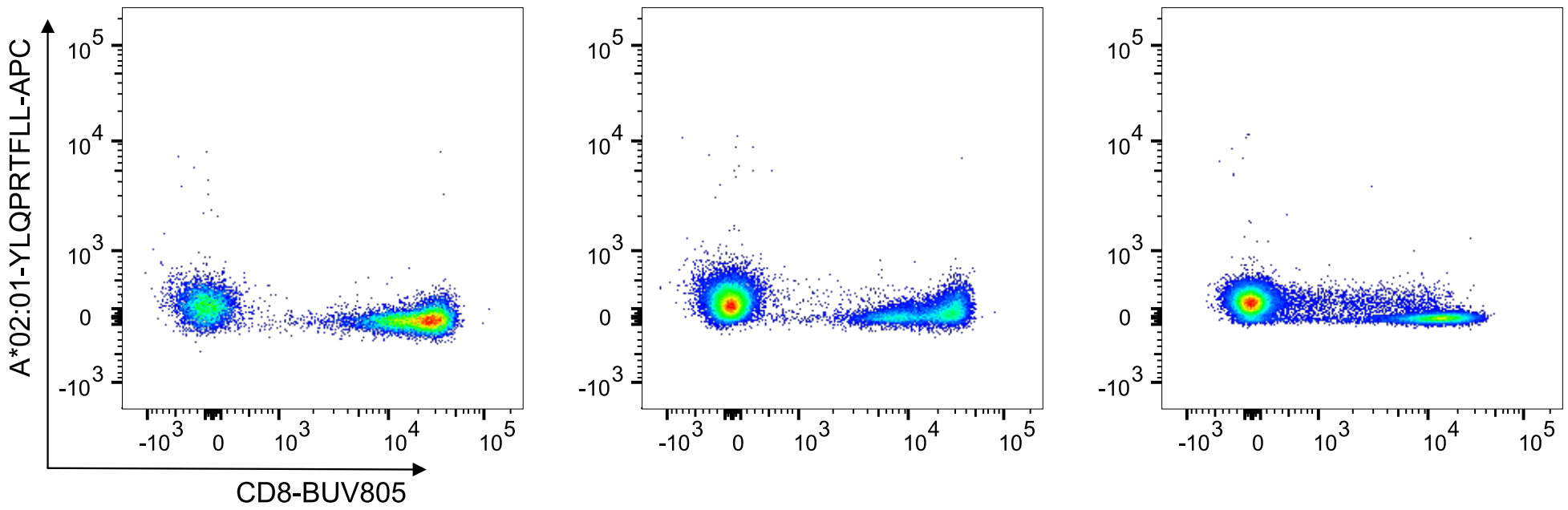

Supplementary Figure 5. To confirm the specificity of the tetramer staining, three HLA-A*02:01-negative COVID-19 patients were stained with the HLA-A*02:01-YLQPRTFLL tetramer and did not show a tetramer-positive CD8$^+$ T-cell population.

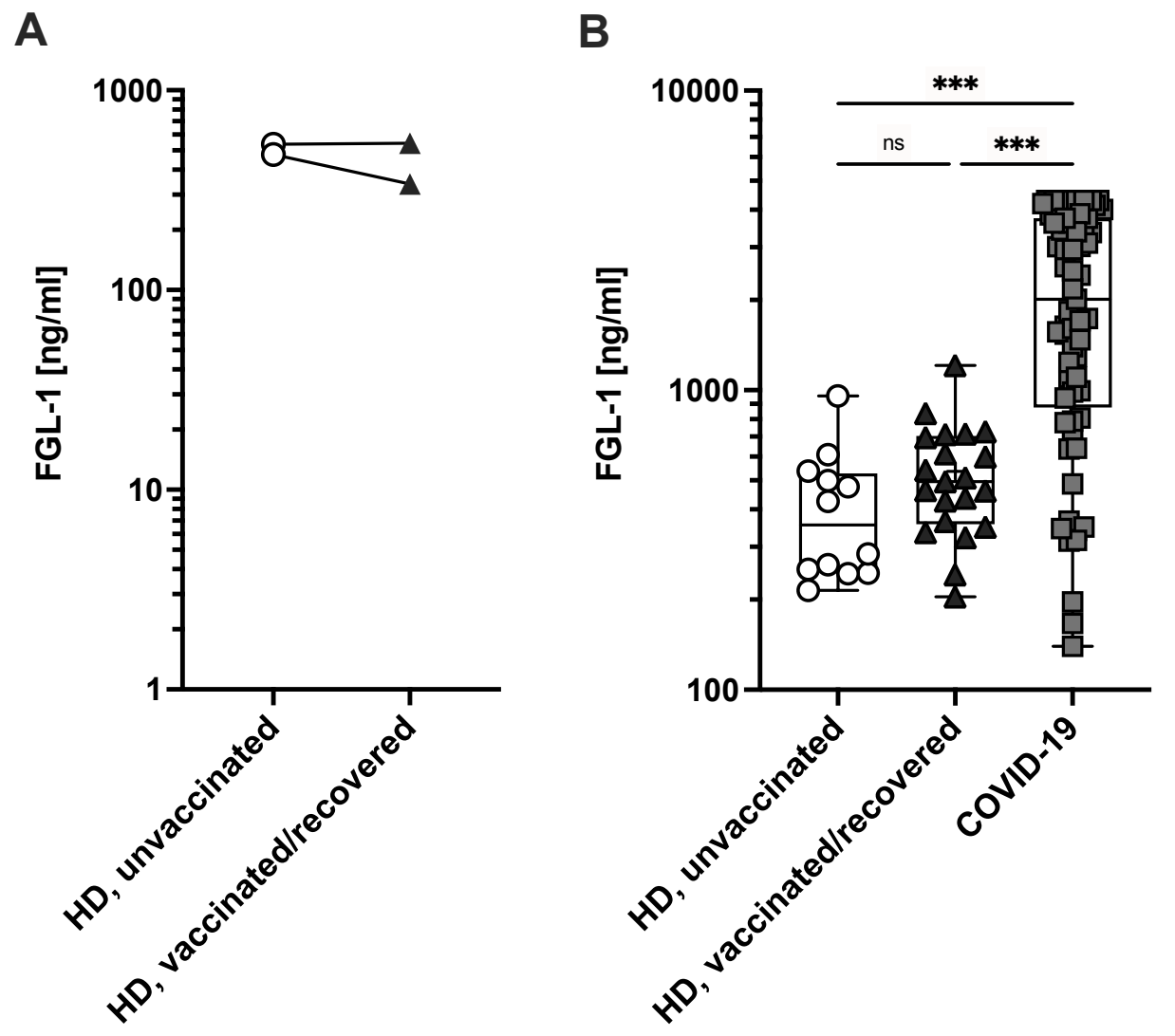

Supplementary Figure 6. (**A**) Plasma FGL-1 levels before COVID-19 vaccination or SARS-CoV-2 infection (unvaccinated) and 9 months after COVID-19 vaccination (vaccinated/recovered) in two healthy donors (HD). (**B**) Plasma FGL-1 levels in healthy donors (HD) before receiving COVID-19 vaccination or SARS-CoV-2 infection (unvaccinated) and after receiving COVID-19 vaccination or recovered after SARS-CoV-2 infection (vaccinated/recovered) compared to patients with COVID-19. A sample dilution of 1:1000 was used for the ELISA analysis. The data were analyzed using a Mann-Whitney test (≥0.05 (ns), <0.05 (*), <0.01 (**), <0.001 (***)). Data are expressed as median with interquartile range.

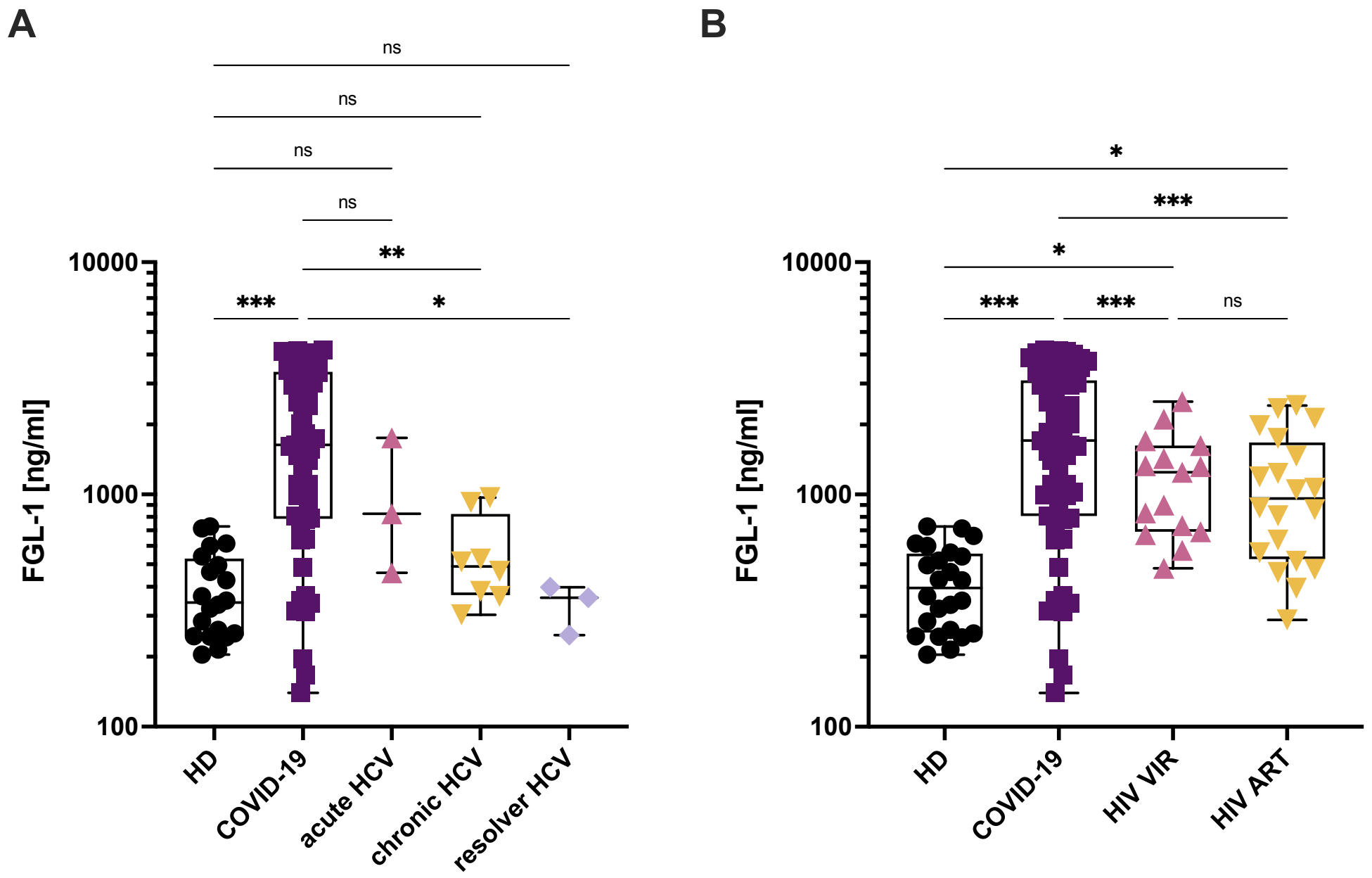

Supplementary Figure 7. Plasma FGL-1 levels in patients with COVID-19 compared to healthy donors (HD), (**A**) patients with HCV, and (**B**) HIV infection. HCV, hepatitis C virus; HIV, human immunodeficiency virus; VIR, Viremic HIV-infected patients; ART, HIV antiretroviral therapy-treated patients. A Mann-Whitney U-test was performed to assess the statistical significance (≥0.05 (ns), <0.05 (*), <0.01 (**), <0.001 (***). Data are expressed as median with interquartile range.

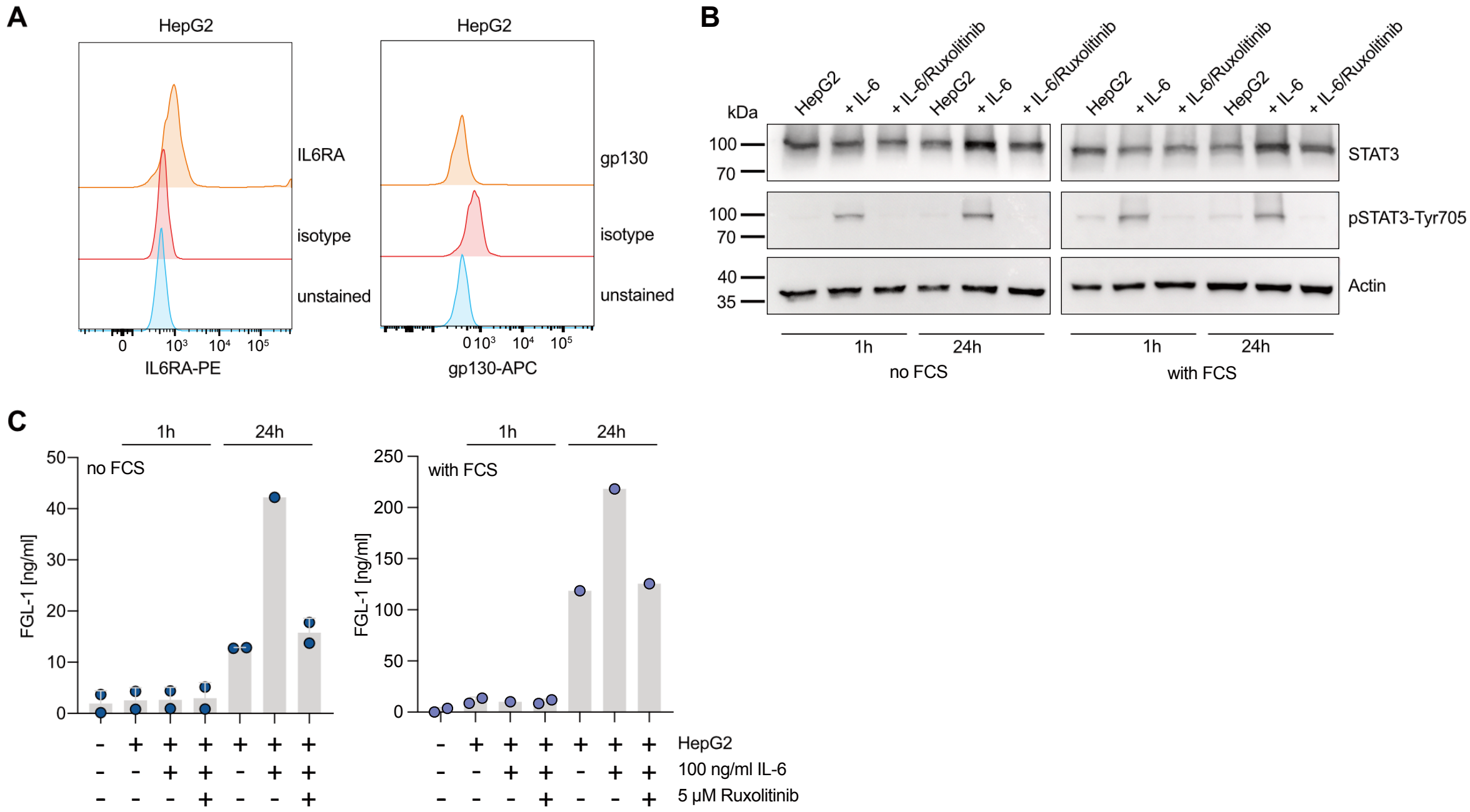

Supplementary Figure 8. IL-6-mediated FGL-1 expression and STAT3 activation in the hepatocyte cell line HepG2. (**A**) Surface staining of the IL-6 receptors IL6Rα (CD126) and gp130 (CD130) on HepG2 cells (**B**) Immunoblot analysis of HepG2 cells to assess IL-6-mediated STAT3 signaling; actin served as a loading control. (**C**) HepG2 cells were cultivated in DMEM F12 medium (10% FCS, 1% penicillin/streptomycin) and serum-free DMEM F12 medium (1% penicillin/streptomycin) and treated with 100 ng/ml IL-6 and 5 μM Ruxolitinib, respectively. Supernatant was collected after 1h and 24h, respectively. The FGL-1 levels were measured by ELISA with a sample dilution of 1:10 and 1:200.

Uncropped immunoblots for Supplementary Figure 8B

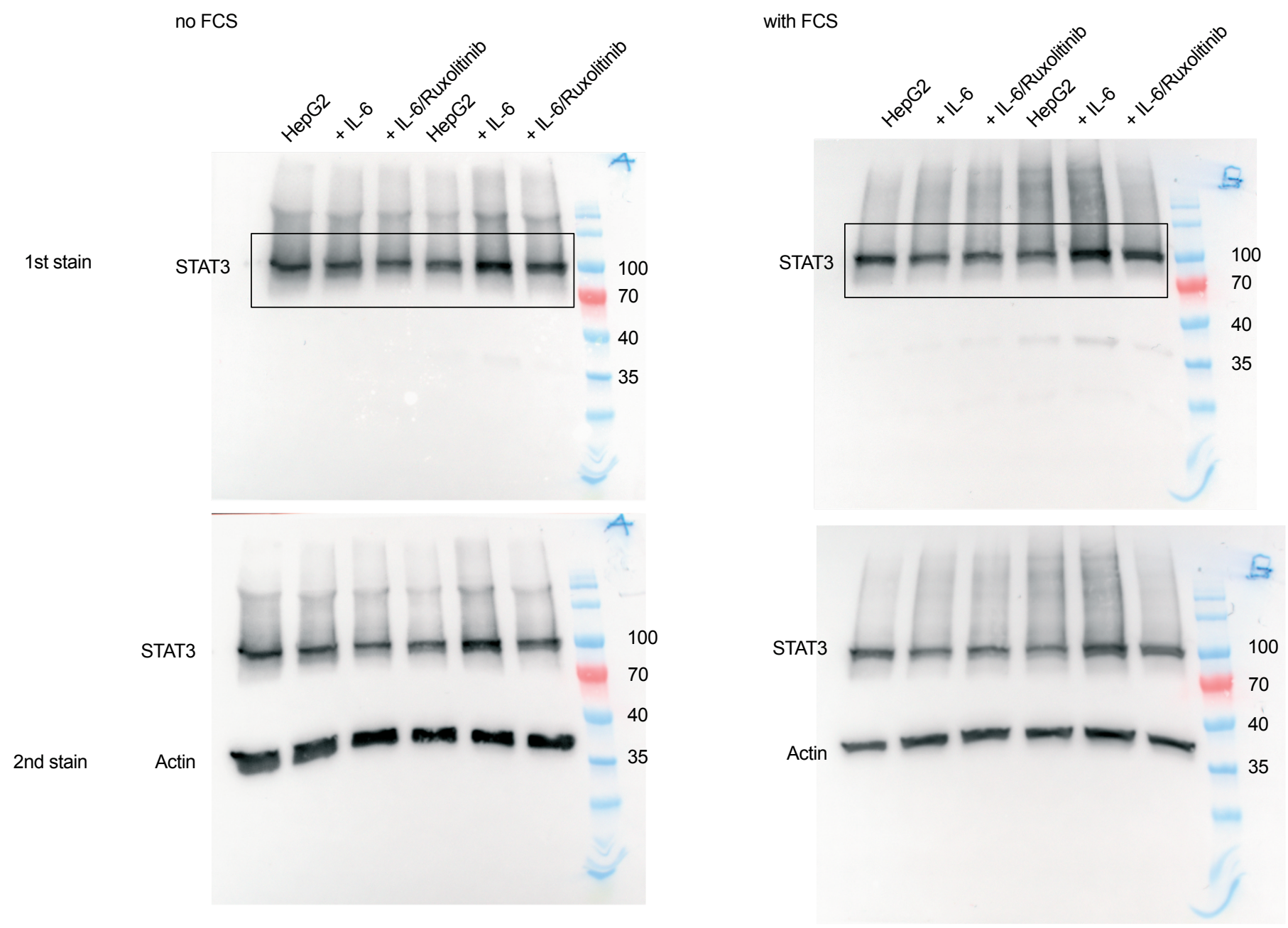

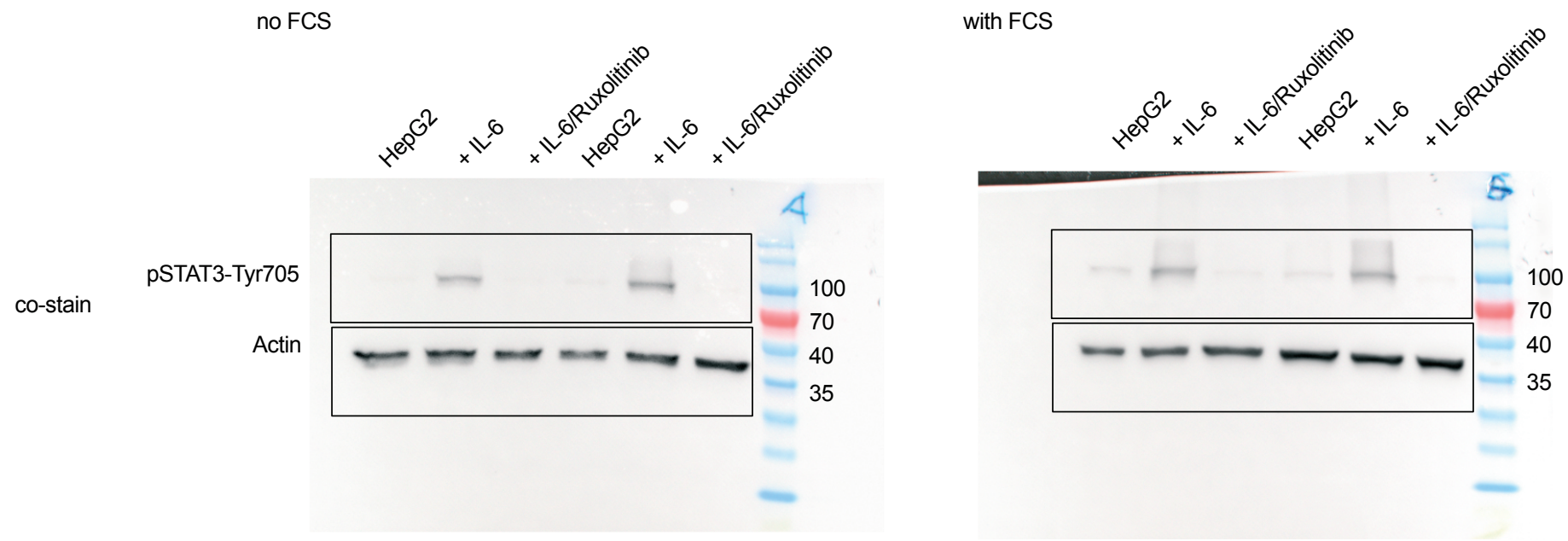

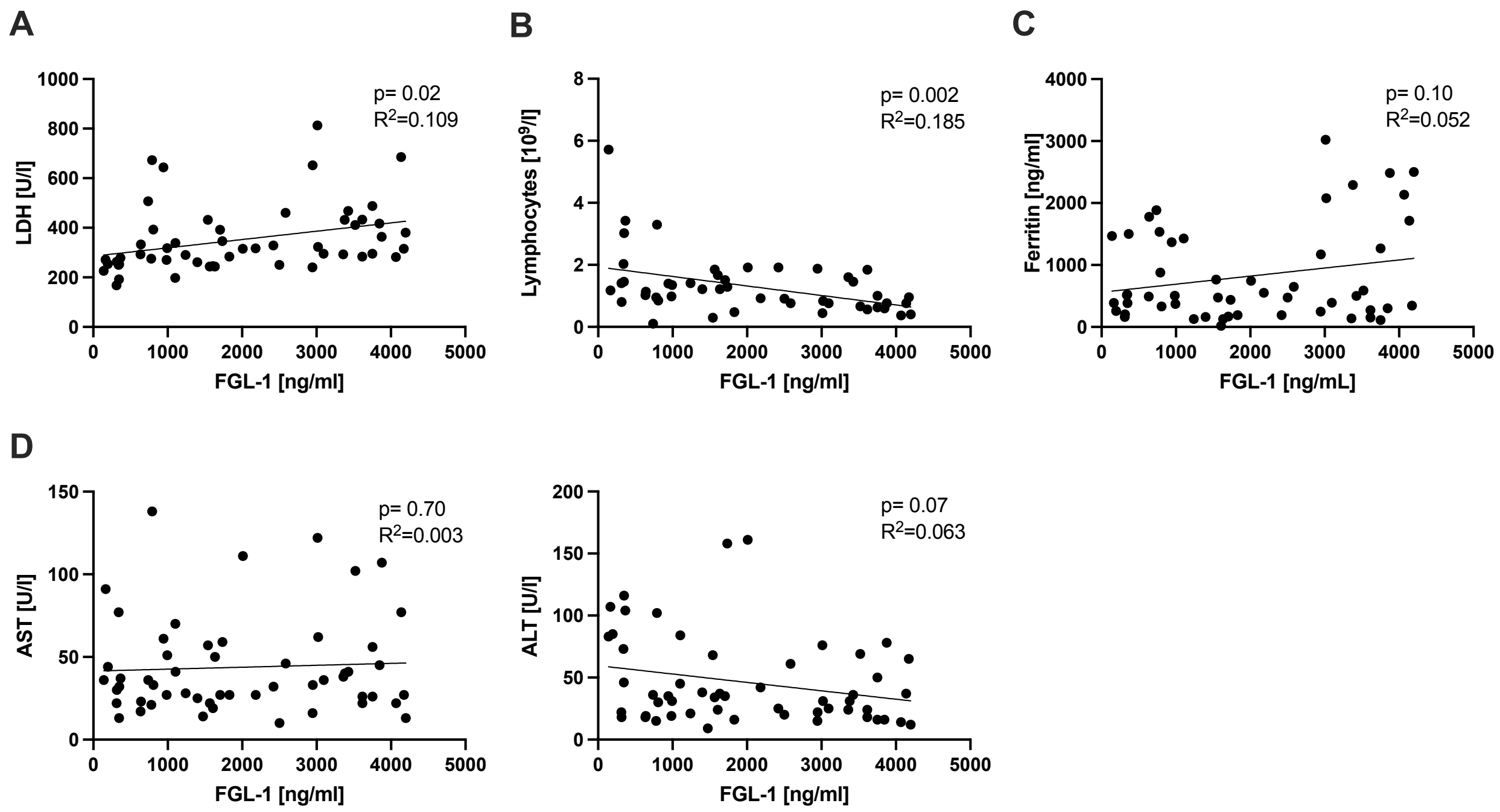

Supplementary Figure 9. Correlation of plasma FGL-1 levels with (**A**) LDH, a marker of cellular damage, (**B**) lymphocyte count, (**C**) ferritin, and (**D**) liver enzymes ALT and AST. For bivariate correlation analysis the Spearman correlation was applied.

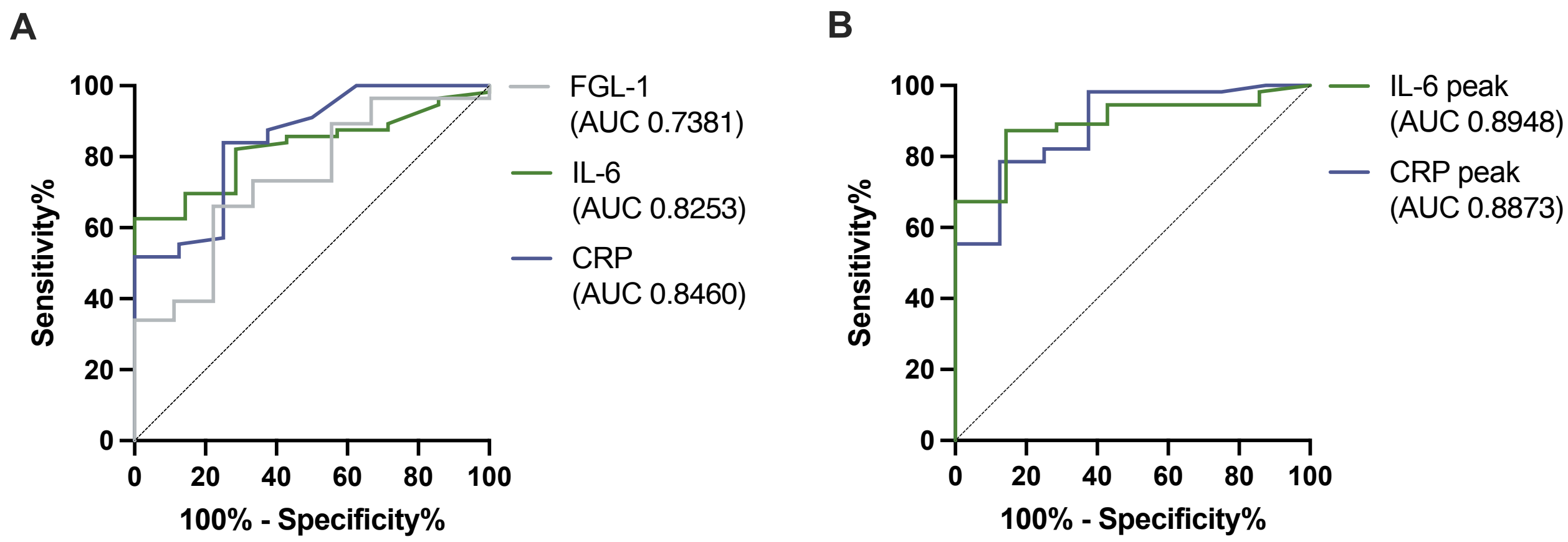

Supplementary Figure 10. Receiver operating characteristic (ROC) curve analysis assessing the ability of FGL-1, CRP, and IL-6 to discriminate between patients with mild and moderate, severe or critical COVID-19. (**A**) ROC curves generated from concurrent measurements of FGL-1, CRP, and IL-6. (**B**) ROC curves based on peak levels of CRP and IL-6.

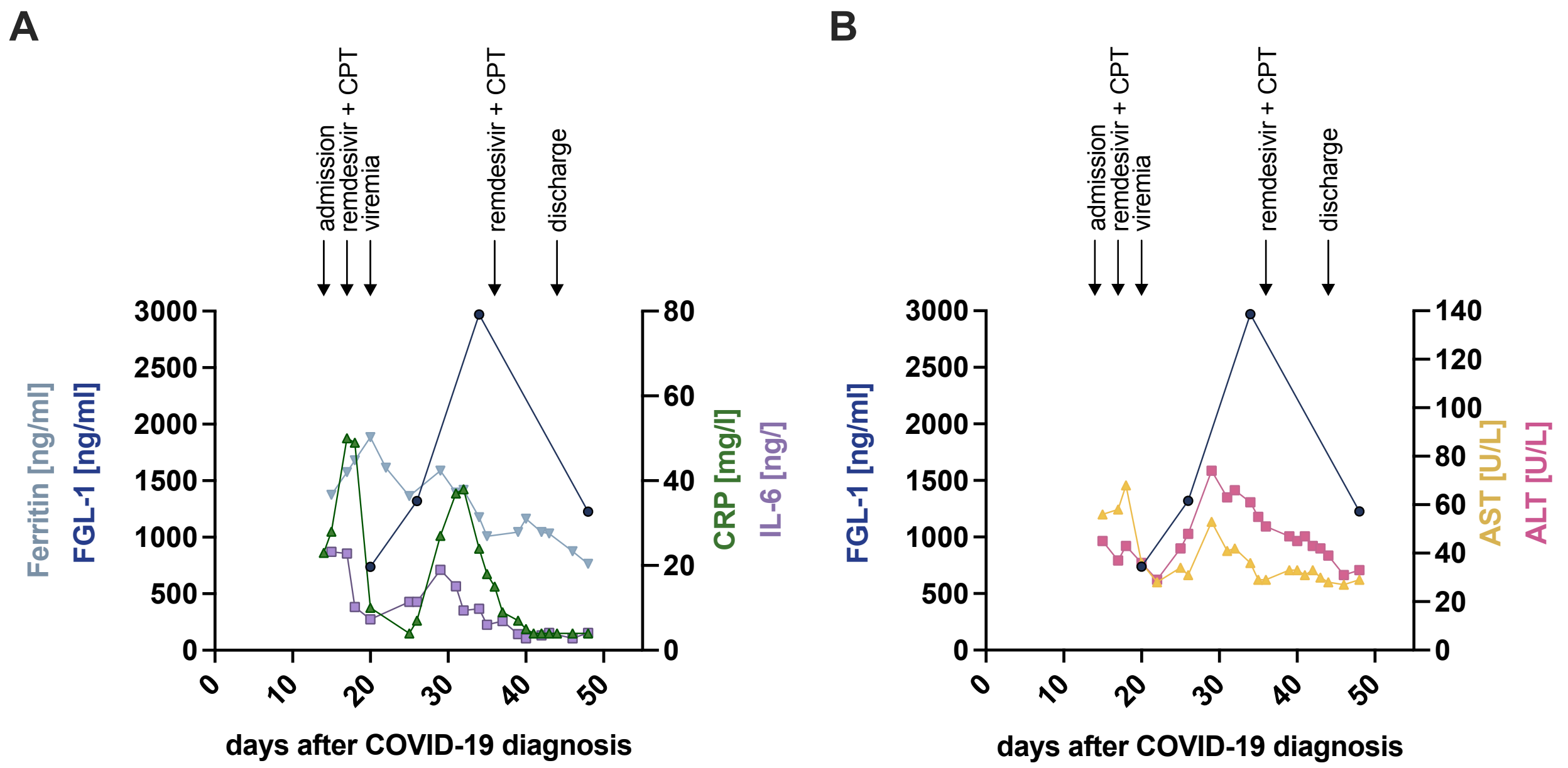

Supplementary Figure 11. Longitudinal assessment of FGL-1 plasma levels and parameters of (**A**) systemic inflammation and (**B**) liver damage during COVID-19 in a patient with severe disease. CRP, C-reactive protein; IL-6, Interleukin-6; AST, aspartate transaminase; ALT, alanine transaminase; CPT, convalescent plasma transfusion.

In December 2020, a 47-year-old woman was diagnosed with SARS-CoV-2 infection when she visited the outpatient oncology department of the University Medical Center Hamburg-Eppendorf for a routine aapointment. A nasopharyngeal swab was taken, and the real-time PCR (RT-PCR) test for SARS-CoV-2 was positive with 19 million copies/ml. Two days before her presentation, mild respiratory symptoms had developed. The patient had a history of stage IIBE refractory primary mediastinal B-cell lymphoma and was in complete remission at the time of SARS-CoV-2 diagnosis after receiving CD19-targeted CAR T-cell therapy (lisocabtagene maraleucel) in April 2020 as part of a study for progressive disease. However, a moderate immunodeficiency with complete peripheral B cell depletion and $CD4^+$ T cells below 500/µL persisted after CAR-T therapy. On the day of admission, day 14 after diagnosis, the patient presented with generalized weakness, cough, lethargy, diarrhea, and dyspnea. The laboratory exams revealed mildly elevated markers of inflammation (CRP 23mg/l; IL-6 23,2ng/l) and mildly elevated liver enzymes (AST 56 U/L; ALT 45 U/L). The patient developed progressive oxygen requirements, and respiratory distress, ultimately requiring noninvasive ventilation. A chest computed tomography scan showed severe pneumonia with pulmonary opacities. Antiviral therapy with remdesivir was initiated for 5 days, together with two transfusions of convalescent plasma (CPT). In the further course, despite therapy, the patient presented with prolonged SARS-CoV-2 virus excretion and viremia. Considering the patient's B cell depletion following CAR-T-cell therapy and persistent T-cell deficiency, prolonged antiviral therapy with remdesivir was administered for an additional 10 days, accompanied by CPT therapy on days 36, 38, 40, 42, and 44 after diagnosis. After the patient's condition was stabilized, she was discharged on day 50 after diagnosis. Although SARS-CoV-2 was still detectable in nasopharyngeal swabs, viremia had subsided. While inflammatory markers normalized, liver enzyme elevations, potentially indicating liver involvement in SARS-CoV-2 infection, peaked later, reaching a grade 2 liver injury according to Common Terminology Criteria for Adverse Events (AST, 92 U/L; ALT, 126 U/L). The elevated liver enzymes gradually normalized over time without intervention.

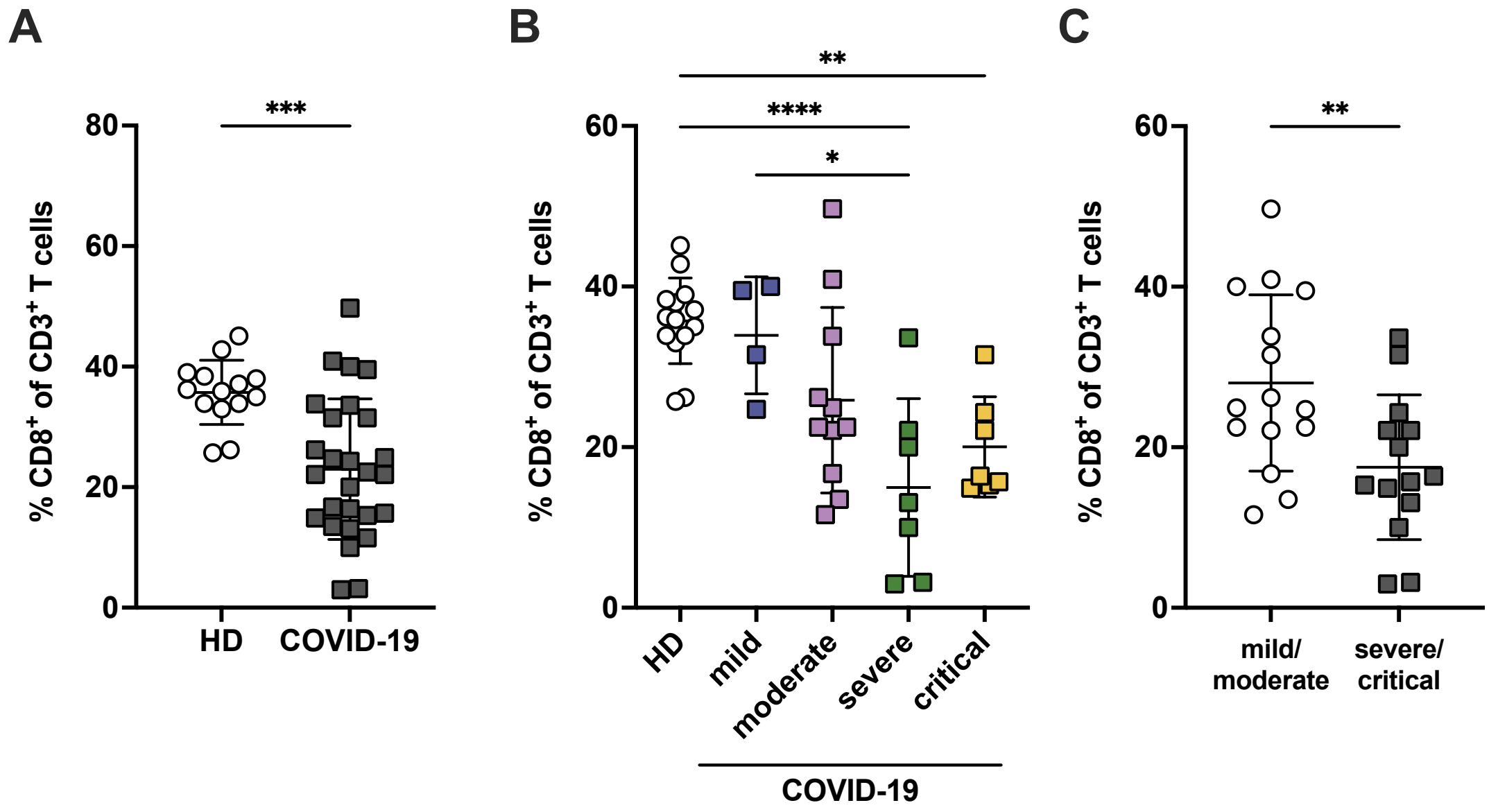

Supplementary Figure 12. (**A**) Frequencies of CD8⁺ T cells among CD3⁺ T cells in patients with COVID-19 (n = 27) compared to healthy donors (HD, n = 14). (**B, C**) Frequencies of CD8⁺ T cells among CD3⁺ T cells in patients with COVID-19 with mild, moderate, severe, and critical disease. A Mann-Whitney U-test (A, C) and and Kruskal-Wallis test was performed to assess the statistical significance (≥0.05 (ns), <0.05 (*), <0.01 (**), <0.001 (***). Data are expressed as median with interquartile range.

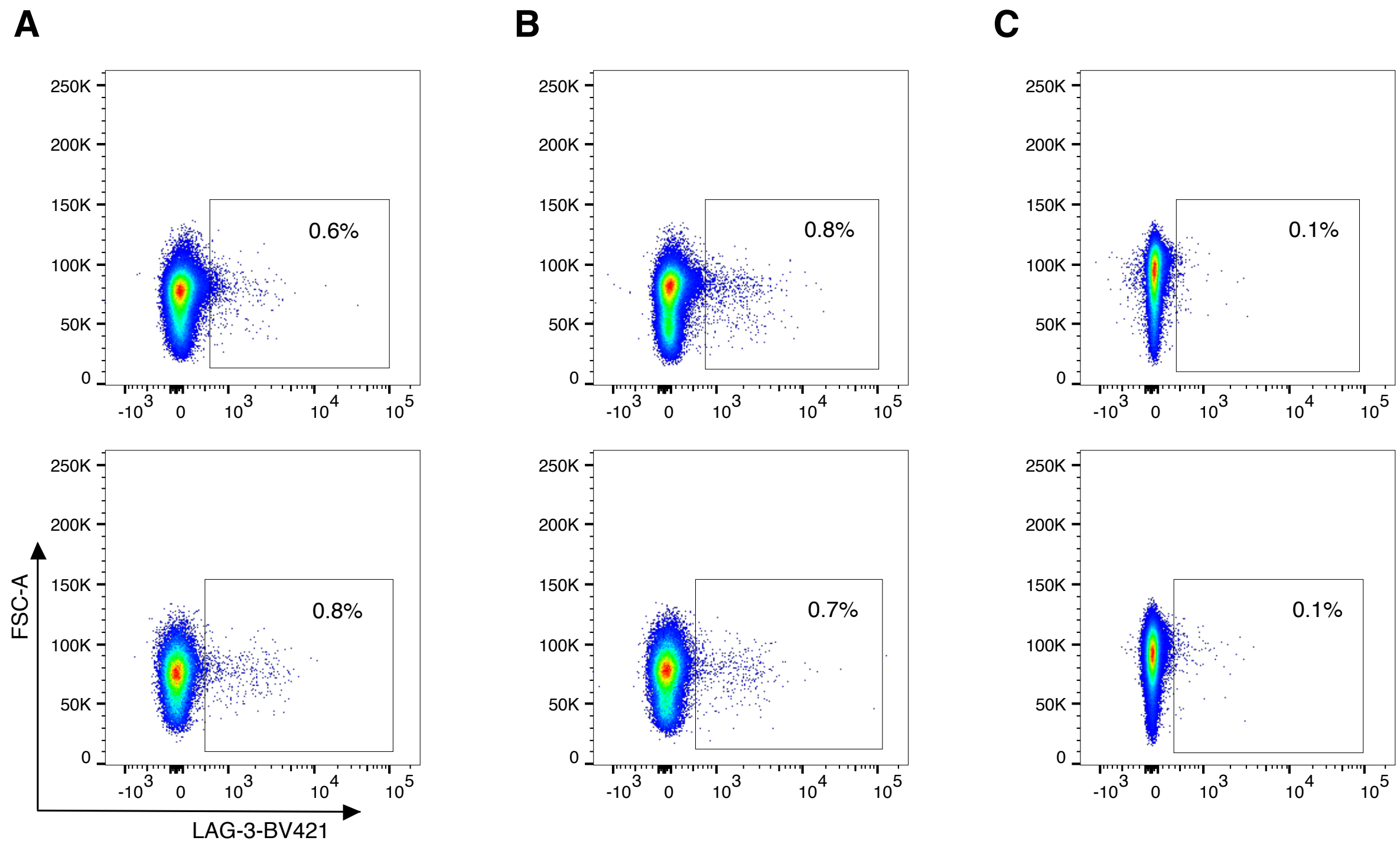

Supplementary Figure 13. Representative dot plots of two healthy donors showing the LAG-3 expression of CD8⁺ T cells at a time point (**A**) before receiving COVID-19 vaccination or SARS-CoV-2 infection, (**B**) 9 months after receiving COVID-19 vaccination and (**C**) after SARS-CoV-2 infection.

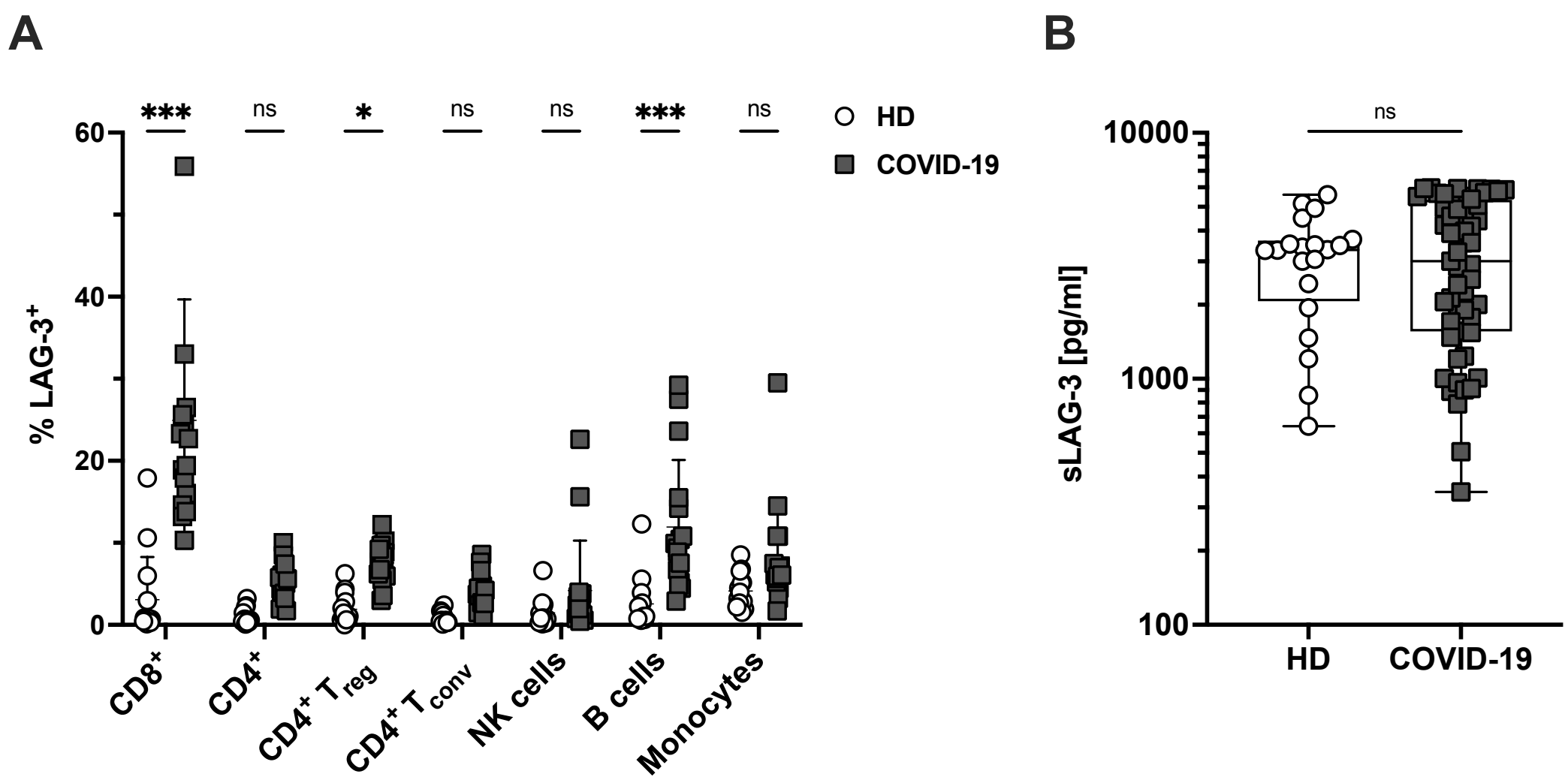

Supplementary Figure 14. (**A**) LAG-3 expression among all immune cell subsets in patients with COVID-19 (n = 16) compared with healthy donors (HD, n = 14). (**B**) Plasma levels of soluble LAG-3 (sLAG-3) in patients with COVID-19 compared to HD. Natural killer cells, NK cells; T regulatory cells, T_{reg}; T conventional cell, T_{conv}. A Mann-Whitney U-test and two-way ANOVA test was performed to assess the statistical significance (≥0.05 (ns), <0.05 (*), <0.01 (**), <0.001 (***)). Data are expressed as median with interquartile range.

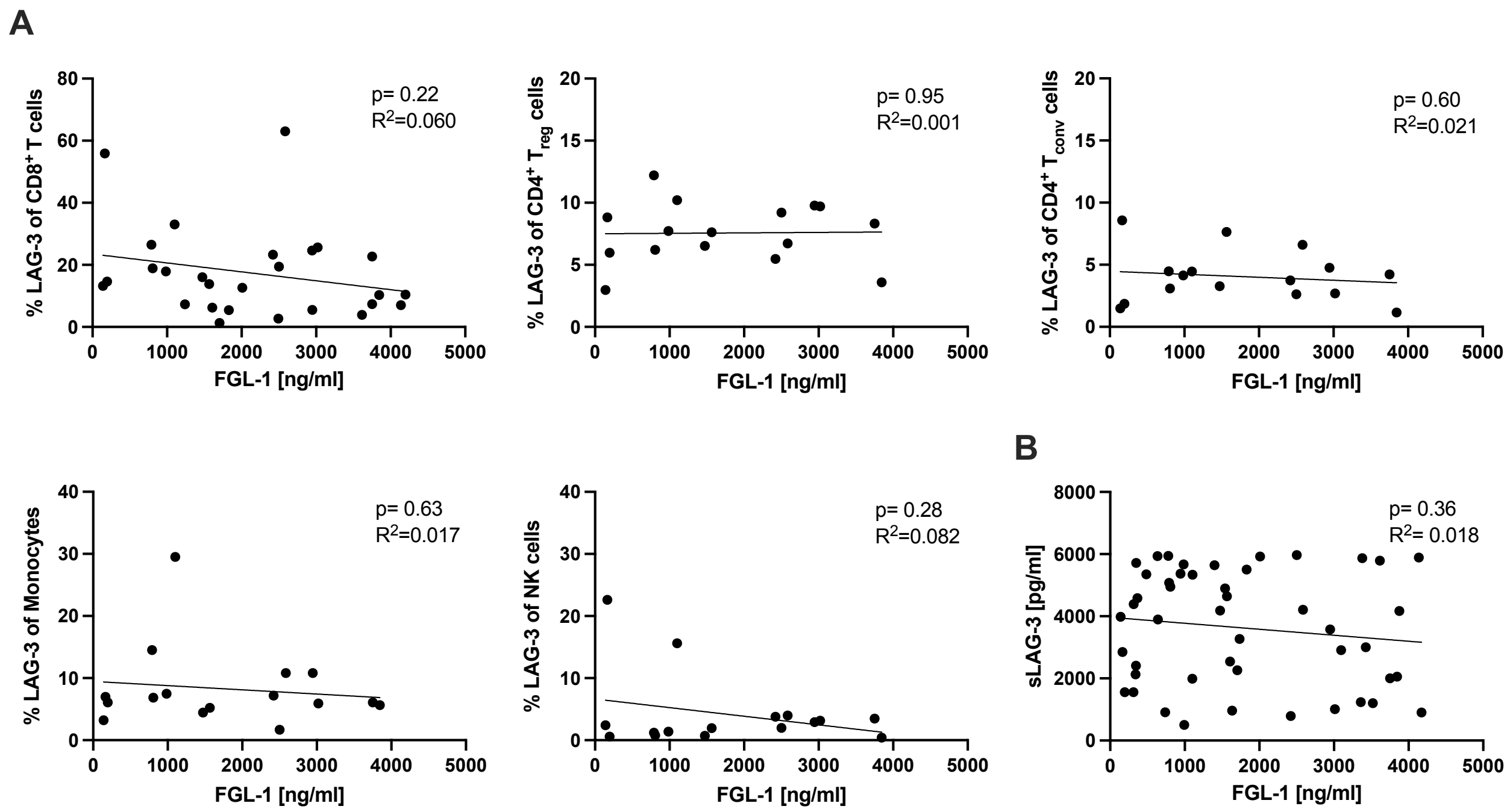

Supplementary Figure 15. Correlation of plasma FGL-1 levels with (**A**) the frequency of LAG-3$^+$ cells of any immune subset and (**B**) with soluble LAG-3 in plasma samples of COVID-19 patients. Natural killer cells, NK cells; T regulatory cells, T_{reg}; T conventional cell, T_{conv}. For bivariate correlation analysis the Spearman correlation was applied.

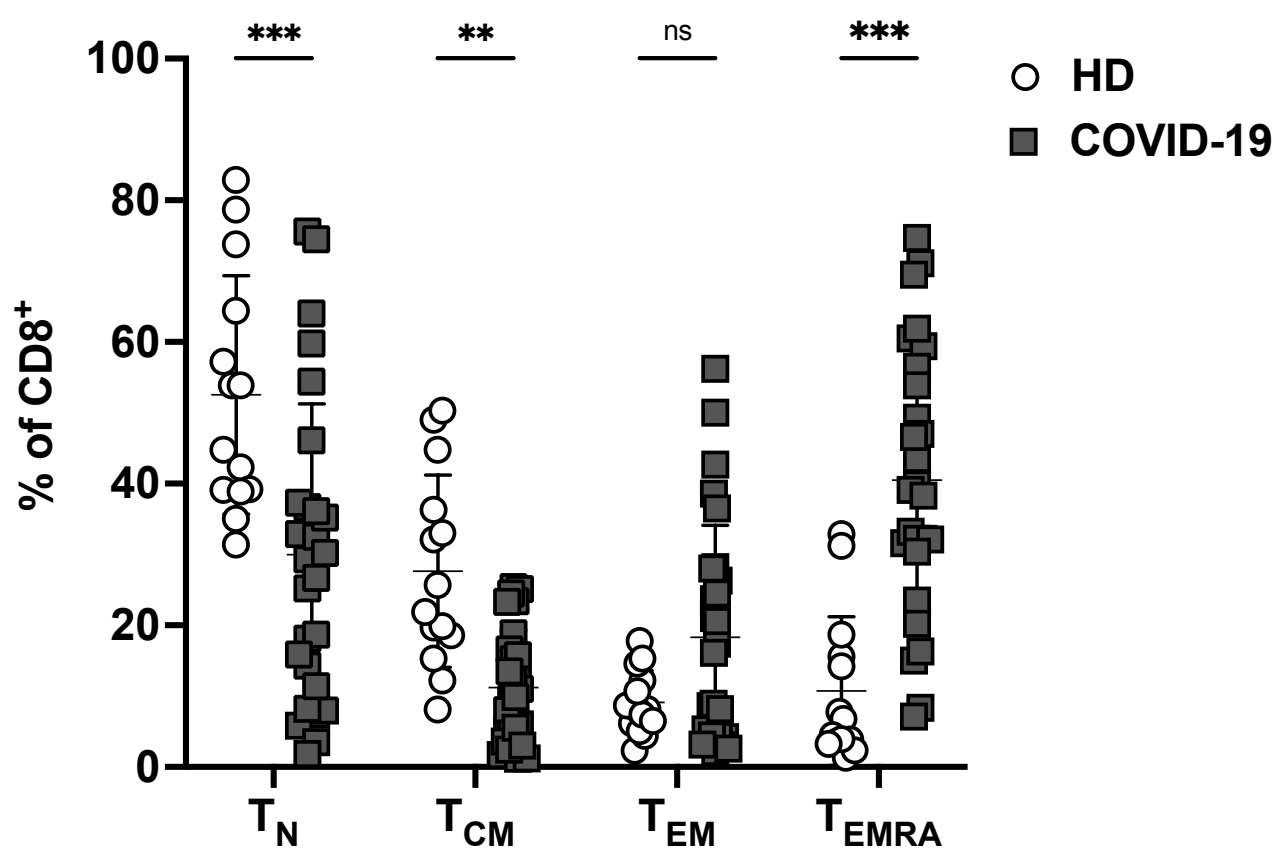

Supplementary Figure 16. Frequency of T_N, T_{CM}, T_{EM}, and T_{EMRA} $CD8^+$ T cells in patients with COVID-19 (n = 27) compared to healthy donors (HD, n = 14). T_N, naïve; T_{CM}, central memory; T_{EM}, effector memory; T_{EMRA}, terminally differentiated effector memory cells. A two-way ANOVA test was performed to assess the statistical significance (≥0.05 (ns), <0.05 (*), <0.01 (**), <0.001 (***)). Data are expressed as median with interquartile range.

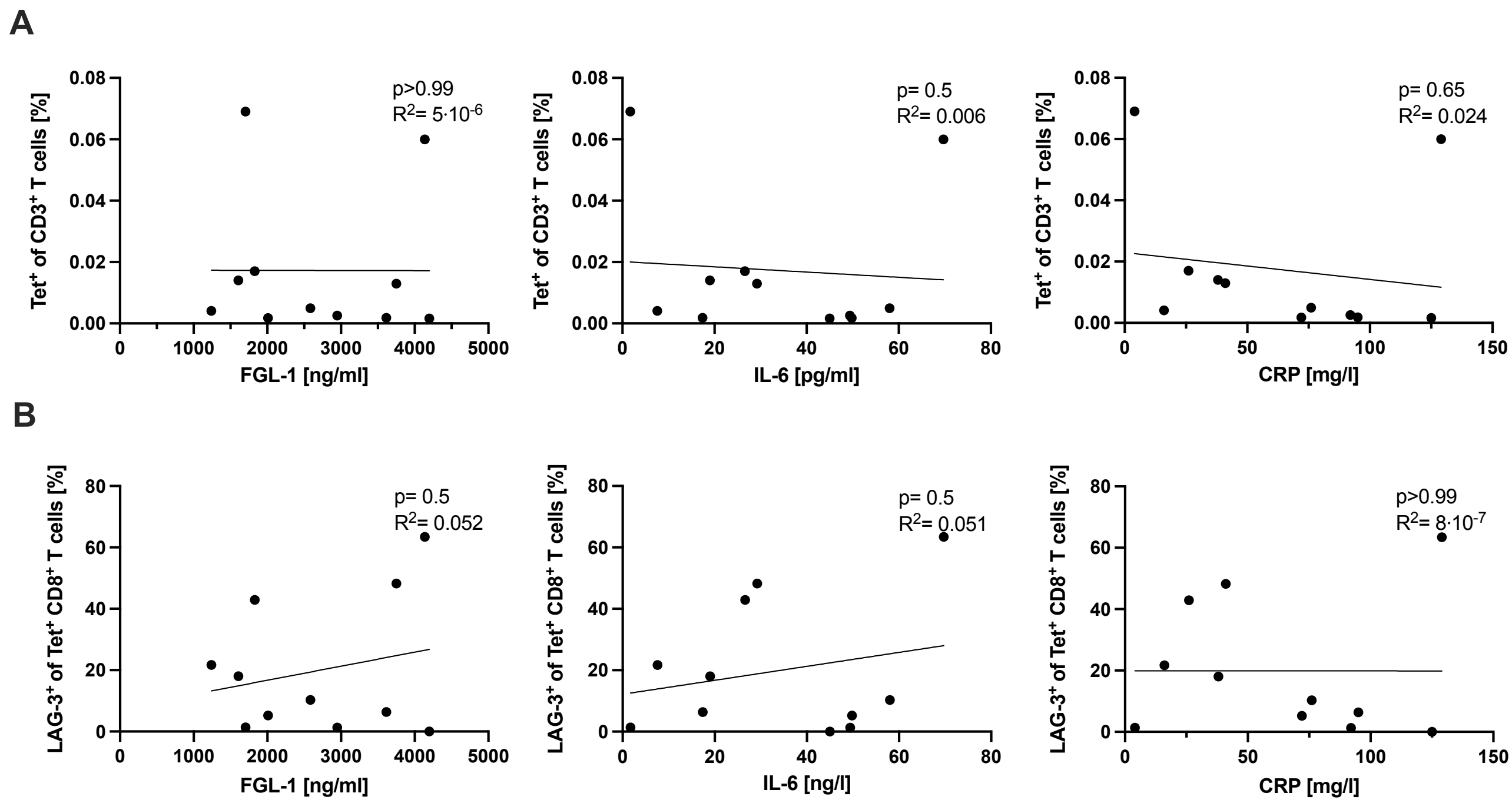

Supplementary Figure 17. (**A**) Correlations between the ex vivo frequency of SARS-CoV-2-specific CD8+ T cells in relation to FGL-1, IL-6, and CRP levels. (**B**) Correlations between the LAG-3+ expression on SARS-CoV-2-specific CD8+ T cells and FGL-1, IL-6, and CRP levels. Tet+, tetramer-positive SARS-CoV-2-specific CD8+ T cells. P-values were calculated by the Spearman correlation for bivariate correlations.

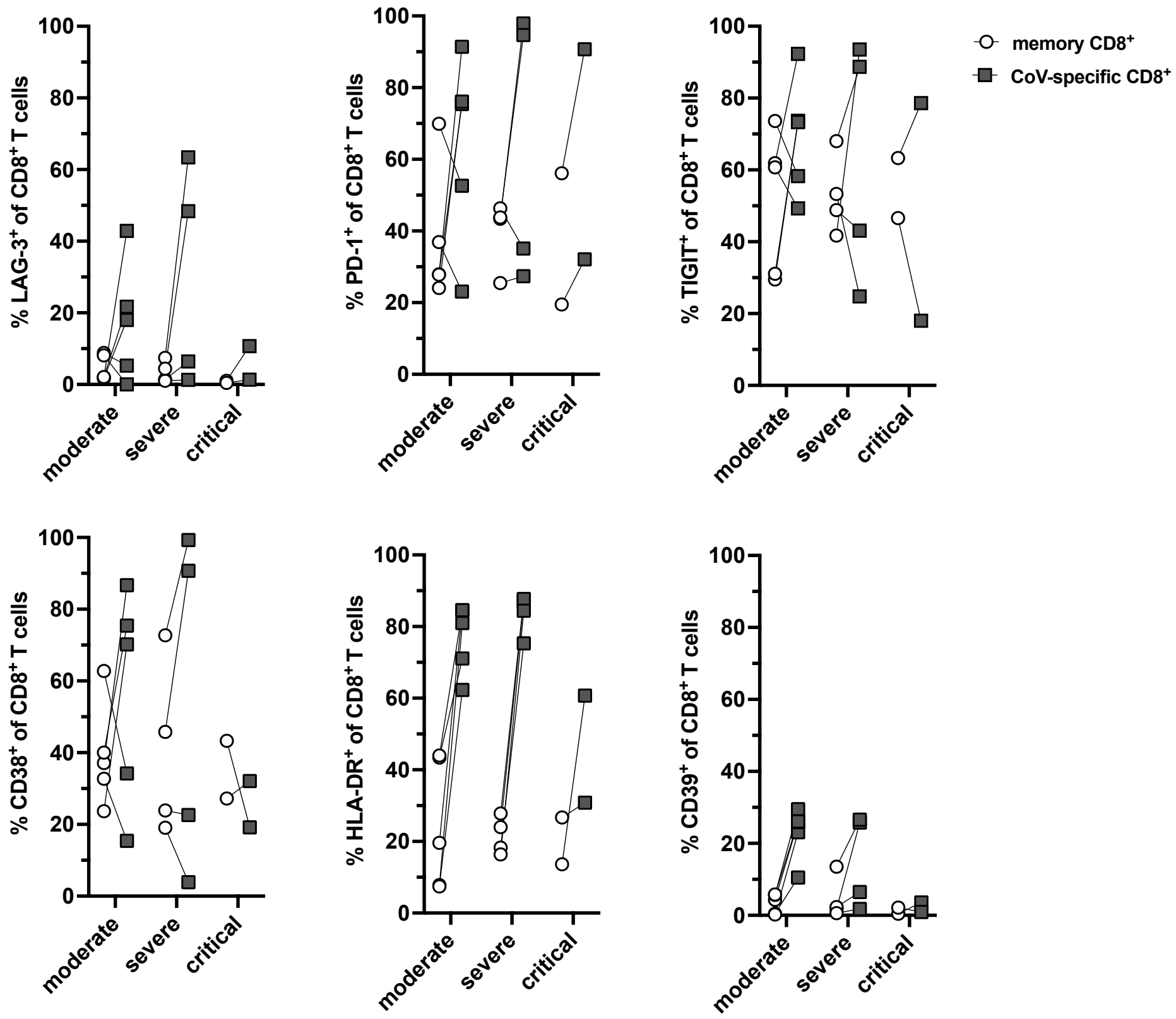

Supplementary Figure 18. Frequencies of LAG-3⁺, PD-1⁺, TIGIT⁺, CD38⁺, HLA-DR⁺, and CD39⁺ cells of SARS-CoV-2-specific CD8⁺ T cells and CD8⁺ memory T cells in patients with COVID-19 with moderate, severe, and critical disease. Due to the limited sample size in the subgroups, no statistical tests were performed.

Supplementary Methods

Immunoblotting

HepG2 cells (DSMZ, Braunschweig, Germany) were cultured in DEMEM F12 supplemented with 10% (v/v) fetal calf serum (FCS) and 1% (v/v) penicillin/streptomycin. For treatment with IL-6 (BioLegend) and/or ruxolitinib (JAK1/2 inhibitor; Selleck Biotechnology), $1x10^6$ cells were seeded in 6-wells with or without FCS and treated for 1 or 24h with 100 ng/ml IL-6 or 100 ng/ml IL-6 + 5 µM ruxolitinib. Supernatants were harvested and stored at -20°C for FGL-1 quantification and whole cell extracts were generated using radioimmunoprecipitation assay buffer (Thermo Fisher Scientific) supplemented with protease and phosphatase inhibitors (Roche, Basel, Switzerland). After quantification using Bradford assay (Thermo Fisher Scientific), 20 µg extract were separated on denaturing 10% Mini-PROTEAN Precast Gels (Bio-Rad). Proteins were plotted under semi-dry conditions using Trans-Blot Turbo System with the PVDF Transfer Packs (Bio-Rad). Blocking was performed with AdvanBlock-Chemi (Advansta, San Jose CA, US). Signals were visualized by chemiluminescence using WesternBright ECL (Advansta) and read out on a Vilber Fusion FX. Antibodies used: anti-STAT3 (clone 124H6, dilution 1:2000, #9139, Cell Signaling), anti-pSTAT3-Tyr705 (clone D3A7, dilution 1:1000, #9145S Cell Signaling). Secondary antibodies: anti-mouse-HRP (HAF018) and anti-rabbit-HRP (NB7160), bot at 1:5000 (both R&D Technology).

Multiparameter Flow Cytometry analysis of HepG2 cells

For multiparametric flow cytometry analysis, HepG2 cells (DSMZ, Braunschweig, Germany) were stained with an APC-labeled gp130 (CD130)-specific antibody (clone 2E1B02) or PE-labeled IL-6Rα (CD126)-specific antibody (clone UV4) (1:200, BioLegend). Isotype controls included mIgG2a (clone MOPC-173) and mIgG1 (clone MOPC-21), both at 1:200 dilution (BioLegend).

Statistical analyses

All flow cytometric data were analyzed using FlowJo v10.8 software (BD Biosciences, New Jersey, USA). Graphs and plots were created using GraphPad Prism v10.3.1 (GraphPad Software Inc., San Diego, CA, USA). The statistical tests used to assess statistical significance in unpaired and paired datasets are indicated in the respective figure legends. Spearman's rank correlation coefficient was calculated to determine the correlation between different data sets. Data are expressed as mean with standard deviation. Multiple linear regression analysis was performed in GraphPad Prism to examine the relationship between FGL-1 levels and the predictors age, sex, disease severity, comorbidities (any), lymphocyte count, IL-6, CRP, LDH, ALT and ferritin levels. Multicollinearity was examined using variance inflation factors. Results are presented as regression coefficients (β) with 95% confidence intervals and corresponding p-values. Receiver operating characteristic (ROC) curve analysis was performed in GraphPad Prism to assess the ability of FGL-1, CRP, and IL-6 to discriminate between patients with mild and moderate, severe or critical COVID-19.
