# Supplementary material for: FGL-1 plasma levels correlate with markers of inflammation and severe disease during SARS-CoV-2 infection
Source: Commun Med (Lond). 2026 Jul 31;6:439. doi: 10.1038/s43856-026-01775-4 (PMC13469107; doi:10.1038/s43856-026-01775-4)
Supplement: Supplementary file 3 [file 43856_2026_1775_MOESM3_ESM.pdf]

Description of Additional Supplementary Files

File name: Supplementary Data 1

Description: Source Data Set in Excel sheet with numerical values underlying all graphs presented in Fig. 1–4 and Supplementary Fig. 1-18
